# Fourier Transform on the Homogeneous Space of 3D Positions and Orientations for Exact Solutions to Linear PDEs

**DOI:** 10.3390/e21010038

**Published:** 2019-01-08

**Authors:** Remco Duits, Erik J. Bekkers, Alexey Mashtakov

**Affiliations:** Department of Mathematics and Computer Science (CASA), Eindhoven University of Technology, 5600 MB Eindhoven, The Netherlands

**Keywords:** fourier transform, rigid body motions, partial differential equations, Lévy processes, Lie Groups, homogeneous spaces, stochastic differential equations

## Abstract

Fokker–Planck PDEs (including diffusions) for stable Lévy processes (including Wiener processes) on the joint space of positions and orientations play a major role in mechanics, robotics, image analysis, directional statistics and probability theory. Exact analytic designs and solutions are known in the 2D case, where they have been obtained using Fourier transform on SE(2). Here, we extend these approaches to 3D using Fourier transform on the Lie group SE(3) of rigid body motions. More precisely, we define the homogeneous space of 3D positions and orientations $R^{3}⋊S^{2}:=SE\left(3\right)/\left(\left\{0\right\}\times SO\left(2\right)\right)$ as the quotient in SE(3). In our construction, two group elements are equivalent if they are equal up to a rotation around the reference axis. On this quotient, we design a specific Fourier transform. We apply this Fourier transform to derive new exact solutions to Fokker–Planck PDEs of α-stable Lévy processes on $R^{3}⋊S^{2}$. This reduces classical analysis computations and provides an explicit algebraic spectral decomposition of the solutions. We compare the exact probability kernel for α=1 (the diffusion kernel) to the kernel for $\alpha =\frac{1}{2}$ (the Poisson kernel). We set up stochastic differential equations (SDEs) for the Lévy processes on the quotient and derive corresponding Monte-Carlo methods. We verified that the exact probability kernels arise as the limit of the Monte-Carlo approximations.

## 1. Introduction

The Fourier transform has had a tremendous impact on various fields of mathematics including analysis, algebra and probability theory. It has a broad range of applied fields such as signal and image processing, quantum mechanics, classical mechanics, robotics and system theory. Thanks to Jean-Baptiste Joseph Fourier (1768–1830), who published his pioneering work “Théory analytique de la chaleur” in 1822, the effective technique of using a Fourier transform to solve linear PDE-systems (with appropriate boundary conditions) for heat transfer evolutions on compact subsets Ω of $R^{d}$ was born. The Fourier series representations of the solutions helped to understand the physics of heat transfer. Due to the linearity of the evolution operator that maps the possibly discontinuous square integrable initial condition to the square integrable solution at a fixed time t>0, one can apply a spectral decomposition which shows how each eigenfunction is dampened over time. Thanks to contributions of Johann Peter Gustav Lejeune Dirichlet (1805–1859), completeness of the Fourier basis could then be formalized for several boundary conditions. Indeed, separation of variables (also known as “the Fourier method”) directly provides a Sturm–Liouville problem [1] and an orthonormal basis of eigenfunctions for $L_{2}\left(\Omega \right)$, which is complete due to compactness of the associated self-adjoint kernel operator. When dilating the subset Ω to the full space $R^{d}$, the discrete set of eigenvalues start to fill R and the discrete spectrum approximates a continuous spectrum (see, e.g., [2]). Then, a diffusion system on $R^{d}$ can be solved via a unitary Fourier transform on $L_{2}\left(R^{d}\right)$ (cf. [3]).

Nowadays, in fields such as mechanics/robotics [4,5,6,7], mathematical physics/harmonic analysis [8], machine learning [9,10,11,12,13] and image analysis [14,15,16,17,18,19], it is worthwhile to extend the spatial domain of functions on $M=R^{d}$ (or $M=Z^{d}$) to groups G=M⋊T that are the semi-direct product of an Abelian group *M* and another matrix group *T*. This requires a generalization of the Fourier transforms on the Lie group $\left(R^{d},+\right)$ towards the groups $G=R^{d}⋊T$. Then, the Fourier transform gives rise to an invertible decomposition of a square integrable function into irreducible representations. This is a powerful mechanism in view of the Schur’s lemma [20,21] and spectral decompositions [22,23]. However, it typically involves regularity constraints ([22], ch:6.6, [24], ch:3.6) on the structure of the dual orbits in order that Mackey’s imprimitivity theory [25] can be applied to characterize *all* unitary irreducible representations (UIRs) of *G*. This sets the Fourier transform on the Lie group *G* [22,24,26]. Here, we omit technicalities on regularity constraints on the dual orbits and the fact that *G* may not be of type I (i.e., the quasi-dual group of *G* may not be equal to the dual group of *G* (cf. [22], thm.7.6, 7.7, [24], ch:3, [27]), as this does not play a role in our case of interest.

We are concerned with the case $M=R^{3}$ and T=SO(3) where G=SE(3)=M⋊SO(3) is the Lie group of 3D rigid body motions. It is a (type I) Lie group with an explicit Fourier transform $F_{G}$ where the irreducible representations are determined by regular dual orbits (which are spheres in the Fourier domain indexed by their radius p>0) and an integer index s∈Z (cf. [4,26]).

In this article, we follow the idea of Joseph Fourier: we apply the Fourier transform $F_{G}$ on the rigid body motion group G=SE(3) to solve both non-degenerate and degenerate (hypo-elliptic) heat flow evolutions, respectively, on the Lie group *G*. More precisely, we design a Fourier transform $F_{G/H}$ on the homogeneous space of positions and orientations G/H with H≡{0}×SO(2) to solve degenerate and non-degenerate heat flow evolutions on the homogeneous space G/H. We also simultaneously solve related PDEs (beyond the diffusion case), as we explain below. For general Fourier theory and harmonic analysis on homogeneous spaces, see the works by Ghaani Farashahi [28,29,30,31], of which the work in [31] applies to our setting $G/H=R^{3}⋊S^{2}$. In contrast to ([31], ch:5.2), we consider the subgroup H≡{0}×SO(2) instead of {0}×SO(3), and we include an extra projection in our design of $F_{G/H}$.

The idea of applying Fourier transforms to solve linear (degenerate) PDEs on non-commutative groups of the type $R^{d}⋊T$ is common and has been studied by many researchers. For example, tangible probability kernels for heat transfer (and fundamental solutions) on the Heisenberg group were derived by Gaveau [32]. They can be derived by application ([23], ch:4.1.1) of the Fourier transform on the Heisenberg group ([22], ch:1). This also applies to probability kernels for degenerate, hypo-elliptic diffusions on $SE\left(2\right)=R^{2}⋊SO\left(2\right)$, where three different types (a Fourier series, a rapidly decaying series, and a single analytic formula that equals the rapidly decaying series) of explicit solutions to probability kernels for (convection-)diffusions were derived in previous works by Duits et al. [33,34,35,36]. For a concise review, see ([37], ch:5.1). Here, the two fundamental models for contour perception by, respectively, Mumford [38], Petitot [39] and Citti and Sarti [15] formed great sources of inspiration to study the degenerate diffusion problem on SE(2).

The degenerate (hypo-elliptic) diffusion kernel formula in terms of a Fourier series representation was generalized to the much more wide setting of unimodular Lie groups by Agrachev, Boscain, Gauthier and Rossi [23]. This approach was then pursued by Portegies and Duits to achieve explicit exact solutions to (non-)degenerate (convection-)diffusions on the particular SE(3) case (see [40]).

The idea of using Fourier transform on SE(3) to represent solutions to the linear heat equations on SE(3) has been considered by other authors in a wide variety of applications in the last decade. For a concise theoretical survey, see the recent work of Chirikjian [41]; for related articles with convincing applications, see [42,43]. In the recent work by Portegies and Duits [40], exact solutions are expressed in terms of an explicit, converging, eigenfunction decomposition in spheroidal wave-functions via technical, classical analysis techniques. This provides exact, analytic and converging series expressions that hold (and allow for analysis) prior to any numerical approximation. They can be used to compare different numerical techniques, as was done by Zhang and Duits et al. in the SE(2) case [37]. In numerical implementations, the exact series must be truncated, and, as the spectrum is derived analytically, it is easy to control and reduce approximation errors to a neglectable level [44] (as in the SE(2)-case ([37], ch:5.1.4, thm 5.2 and 5.3, ch:6) with comparisons to an alternative single formula by Duits ([36], thm 5.2)).

Here, we aim to simplify and generalize the explicit spectral decompositions [40] of degenerate diffusions on $R^{3}⋊S^{2}=SE\left(3\right)/H$, and to put this in the algebraic context of Fourier transform on G=SE(3) [4,26,41], or more efficiently on the algebraic context of a Fourier transform on G/H. To this end, we first propose a specific Fourier transform on G/H in Theorem 1. Then, we use it to derive explicit spectral decompositions of the evolution operator in Theorem 2, from which we deduce explicit new kernel expressions in Theorem 3. Finally, we generalize the exact solutions to other PDE systems beyond the diffusion case: We simultaneously solve the Forward-Kolmogorov PDEs for α-stable Lévy processes on the homogeneous space of positions and orientations. Next, we address their relevance in the fields of image analysis, robotics and probability theory.

In image analysis, left-invariant diffusion PDEs on SE(3) have been widely used for crossing-preserving diffusion and enhancement of fibers in diffusion-weighted MRI images of brain white matter [45,46,47,48,49,50], or for crossing-preserving enhancements of 3D vasculature in medical images [18]. They extend classical works on multi-scale image representations [51,52,53,54] to Lie groups [55].

In robotics, they play a role via the central limit theorem [56] in work-space generation of robot arms ([4], ch.12) and they appear indirectly in Kalman-filtering on SE(3) for tracking [57], motion planning of robotic devices [42], and camera motion estimation [58].

In probability theory, diffusion systems on Lie groups describe Brownian motions [59,60] and they appear as limits in central limit theorem on Lie groups [56].

Both in probability theory [61] and in image analysis [62,63,64,65], the spectral decomposition of the evolution operator also allows simultaneously dealing with important variants of the diffusion evolution. These variants of the heat-evolution are obtained by taking fractional powers $-{\left(-\Delta \right)}^{\alpha }$ (cf. [66]) of the minus Laplacian operator Δ=div∘grad that generates the heat flow (due to Fick’s law and the Gauss divergence theorem), where α∈(0,1].

This generalization allows for heavy tailed distributions of α-stable Lévy processes, which arise in a fundamental generalization [61] of the central limit theorem *where one drops the finite variance condition*. Here, we note that recently an extension of the central limit on linear groups (such as SE(3)) has been achieved for finite second-order moments [56]. In engineering applications, where (iterative group-)convolutions are applied ([4], ch.12 and 13, [9,12,13,67,68,69,70,71]), the “kernel width” represents the spread of information or the scale of observing the signal. In the case the applications allow for an underlying probabilistic model with finite variances, variance is indeed a good measure for “kernel width”. However, often this is not the case. Probability kernels for stochastic Lévy processes (used in directional statistics [72], stock market modeling [73], natural image statistics [65]), and modeling of point-spread functions in acquired images (e.g., in spectroscopy [74])) do require distributions with heavier tails than diffusion kernels. Therefore, “full width at half maximum” is a more generally applicable measure for kernel width than variance, as it applies to all α-stable Lévy processes. The probability distributions for α<1 encode a longer range of interaction via their heavy tails and still allow for unlimitedly sharp kernels.

Finally, regarding entropy, we show that for $\alpha \in \{\frac{1}{2},1\}$ we have monotonic increase of entropy $E_{\alpha }\left(t\right)$ over evolution time t>0 of our α-stable Lévy processes. For α=1, one arrives at a diffusion system, and a previous result by Chirikjian on Lie groups [75], also applies to the Lie group quotient $G/H=R^{3}⋊S^{2}$. Thereby, $E_{1}^{\prime }\left(t\right)=\mathrm{trace}\left\{D\cdot F_{1}\left(t\right)\right\}>0$, where $F_{1}\left(t\right)$ is the Fisher information matrix and D is the diffusion matrix. We show that for $\alpha =\frac{1}{2}$ one arrives at a Poisson system where entropy also increases monotonically over time, again relative to a corresponding Fisher matrix. It is also intriguing, from the perspective of geometric theory of information and heat [76], to study optimal entropy on $R^{3}⋊S^{2}$ and (Fourier) Cramér Transforms building on results [77,78] on $R^{n}$. However, such investigations first require a good grip on the spectral decompositions of the PDE-evolution operators for α-stable Lévy processes via a Fourier transform on $R^{3}⋊S^{2}$, which is our primary focus here.

### 1.1. Structure of the Article

The structure of the article is as follows. In the first part of the Introduction, we briefly discuss the history of the Fourier transform, and its generalization to other groups that are the semi-direct product of the translation group and another matrix group, where we provide an overview of related works. Then, we specify our domain of interest—the Fourier transform on the homogeneous space G/H of positions and orientations, which is a Lie group quotient of the Lie group G=SE(3) with a subgroup *H* isomorphic to {0}×SO(2). Then, we address its application of solving PDE systems on G/H, motivated from applications in image analysis, robotics and probability theory.

There are four remaining subsections of the Introduction. In Section 1.2, we provide basic facts on the homogeneous space G/H of positions and orientations and we provide preliminaries for introducing a Fourier transform on G/H. In Section 1.3, we formulate the PDEs of interest on G/H that we solve. In Section 1.4, we formulate the corresponding PDEs on the group *G*. In Section 1.5, we relate the PDE for $\alpha =\frac{1}{2}$ to a Poisson system and quantify monotonic increase of entropy for $\alpha \in \{\frac{1}{2},1\}$. In Section 1.6, we provide a roadmap on the spectral decomposition of the PDE evolutions.

In Section 2, based on previous works, we collect the necessary prior information about the PDEs of interest and the corresponding kernels. We also describe how to extend the case α=1 (the diffusion case) to the general case α∈(0,1].

In Section 3, we describe the Fourier transform on the Lie group SE(3), where we rely on UIRs of SE(3). In particular, by relating the UIRs to the dual orbits of SO(3) and by using a decomposition with respect to an orthonormal basis of modified spherical harmonics, we recall an explicit formula for the inverse Fourier transform.

In Section 4, we present a Fourier transform $F_{G/H}$ on the quotient $G/H=R^{3}⋊S^{2}$. Our construction requires an additional constraint—an input function must be bi-invariant with respect to subgroup *H*, as explained in Remark 3. This extra symmetry constraint is satisfied by the PDE kernels of interest. We prove a theorem, where we present: (1) a matrix representation for the Fourier transform on the quotient; (2) an explicit inversion formula; and (3) a Plancherel formula.

In Section 5, we apply our Fourier transform on the quotient to solve the PDEs of interest. The solution is given by convolution of the initial condition with the specific kernels (which are the probability kernels of α-stable Lévy process). We find the exact formulas for the kernels in the frequency domain relying on a spectral decomposition of the evolution operator (involved in the PDEs). We show that this result can be obtained either via conjugation of the evolution operator with our Fourier transform on $R^{3}⋊S^{2}$ or (less efficiently) via conjugation of the evolution operator with the Fourier transform acting only on the spatial part $R^{3}$. Then, we present a numerical scheme to approximate the kernels via Monte-Carlo simulation and we provide a comparison of the exact solutions and their approximations. Finally, in Section 6, we summarize our results and discuss their applications. In the appendices, we address the probability theory and stochastic differential equations (SDEs) regarding Lévy processes on $R^{3}⋊S^{2}$.

The main contributions of this article are:

- We construct $F_{R^{3}⋊S^{2}}$—the Fourier transform on the quotient $R^{3}⋊S^{2}$, in Equation (44).
- The matrix representations for $F_{R^{3}⋊S^{2}}$, explicit inversion and Plancherel formulas are shown in Theorem 1.
- The explicit spectral decompositions of PDE evolutions for α-stable Lévy process on $R^{3}⋊S^{2}$, in the Fourier domains of both $R^{3}⋊S^{2}$ and $R^{3}$, are shown in Theorem 2; here, the new spectral decomposition in the Fourier domain of $R^{3}⋊S^{2}$ is simpler and involves ordinary spherical harmonics.
- The quantification of monotonic increase of entropy of PDE solutions for α-stable Lévy processes on $R^{3}⋊S^{2}$ for $\alpha \in \{\frac{1}{2},1\}$ in terms of Fisher information matrices is shown in Proposition 1.
- the exact formulas for the probability kernels of α-stable Lévy processes on $R^{3}⋊S^{2}$, in Theorem 3. This also includes new formulas for the heat kernels (the case α=1), that are more efficient than the heat kernels presented in previous work [40].
- Simple formulation and verifications (Monte-Carlo simulations) of discrete random walks for α-stable Lévy processes on $R^{3}⋊S^{2}$ in Proposition 3. The corresponding SDEs are in Appendix A.

### 1.2. Introduction to the Fourier Transform on the Homogeneous Space of Positions and Orientations

Let G=SE(3) denote the Lie group of rigid body motions, equipped with group product:

(1) $$g_{1}g_{2}=\left(x_{1},R_{1}\right)\left(x_{2},R_{2}\right)=\left(R_{1}x_{2}+x_{1},R_{1}R_{2}\right),\;\mathrm{with}g_{k}=\left(x_{k},R_{k}\right)\in G,\;k=1,2.$$

Here, $x_{k}\in R^{3}$ and $R_{k}\in SO\left(3\right)$. Note that $SE\left(3\right)\;=\;R^{3}⋊\;SO\left(3\right)$ is a semi-direct product of $R^{3}$ and SO(3).

**Definition** **1.** *Let B(H) denote the vector space of bounded linear operators on some Hilbert space H.*

*Within the space B(H), we denote the subspace of bounded linear trace-class operators by*

$$B_{2}\left(H\right)=\left\{A:H\to {H\;|\;A\;\mathrm{linear}\;\mathrm{and}\;|\;|\;|A|\;|\;|}^{2}:=\mathrm{trace}\left(A^{*}A\right)<\infty \right\}.$$

**Definition** **2.** *Consider a mapping $\sigma :G\to B(H_{\sigma })$, where $H_{\sigma }$ denotes the Hilbert space on which each $\sigma_{g}$ acts. Then, σ is a Unitary Irreducible Representation (UIR) of G if*

*1.*  *$\sigma :G\to B(H_{\sigma })$ is a homomorphism;*
*2.*  $\sigma_{g}^{-1}=\sigma_{g}^{*}$ for all g∈G; and
*3.*  *there does not exist a closed subspace V of $H_{\sigma }$ other than $\left\{0,H_{\sigma }\right\}$ such that $\sigma_{g}V\subset V$.*

We denote by $\hat{G}$ the dual group of *G*. Its elements are equivalence classes of UIRs, where one identifies elements via $\sigma_{1}∼\sigma_{2}\Leftrightarrow \mathrm{there}\mathrm{exists}a\mathrm{unitary}\mathrm{linear}\mathrm{operator}\upsilon ,s.t.\sigma_{1}=\upsilon \circ \sigma_{2}\circ \upsilon^{-1}.$ Note that G=SE(3) is a unimodular Lie group of type I, which means that the left and right-invariant Haar measure coincide, and that its dual group and its quasi dual group coincide. Thereby it admits a Plancherel theorem [22,24].

**Definition** **3.** *The Fourier transform $F_{G}\left(f\right)={\left(\left(F_{G}f\right)\left(\sigma \right)\right)}_{\sigma \in \hat{G}}$ of a square-integrable, measurable and bounded function f on G is a measurable field of bounded operators indexed by unitary irreducible representations (UIR’s) σ. Now, $\hat{G}$ can be equipped with a canonical Plancherel measure ν and the Fourier transform $F_{G}$ admits an extension unitary operator from $L_{2}\left(G\right)$ to the direct-integral space $\int_{\hat{G}}^{⊕}B_{2}\left(H_{\sigma }\right)d\nu \left(\sigma \right)$. This unitary extension ([22], 4.25) (also known as “Plancherel transform” ([24], thm.3.3.1)) is given by*

(2) $$\begin{array}{c} F_{G}\left(f\right)=\int_{\hat{G}}^{⊕}\hat{f}\left(\sigma \right)\;d\nu \left(\sigma \right),\;\mathrm{with}\\ \hat{f}\left(\sigma \right)=\left(F_{G}f\right)\left(\sigma \right)=\int_{G}f\left(g\right)\;\sigma_{g^{-1}}dg\;\in B_{2}\left(H_{\sigma }\right),\;\mathrm{for}\mathrm{all}\sigma \in \hat{G}, \end{array}$$

*for all $f\in L_{1}\left(G\right)\cap L_{2}\left(G\right)$.*

The Plancherel theorem states that $∥F_{G}{\left(f\right)∥}_{L_{2}\left(\hat{G}\right)}^{2}=\int_{\hat{G}}\left|\;|\;\right|F_{G}{\left(f\right)\left(\sigma \right)|\;|\;|}^{2}d\nu \left(\sigma \right)=\int_{G}{\left|f\left(g\right)\right|}^{2}dg={∥f∥}_{L_{2}\left(G\right)}^{2}$ for all $f\in L_{2}\left(G\right)$, and we have the inversion formula $f=F_{G}^{-1}F_{G}f=F_{G}^{*}F_{G}f$. For details, see [22,24], and, for detailed explicit computations, see [4].

In this article, we constrain and modify the Fourier transform $F_{G}$ on G=SE(3) such that we obtain a suitable Fourier transform $F_{G/H}$ defined on a homogeneous space

(3) $$R^{3}⋊S^{2}:=G/H\mathrm{with}\mathrm{subgroup}H=\left\{0\right\}\times {\mathrm{Stab}}_{SO(3)}\left(a\right)$$

of left cosets, where ${\mathrm{Stab}}_{SO(3)}\left(a\right)=\left\{R\in SO\left(3\right)\;|\;Ra=a\right\}$ denotes the subgroup of SO(3) that stabilizes an a priori reference axis $a\in S^{2}$, say $a=e_{z}={\left(0,0,1\right)}^{T}$. In the remainder of this article, we set this choice $a=e_{z}$.

**Remark** **1.** *Although the semi-direct product notation $R^{3}⋊S^{2}$ is formally not correct as $S^{2}$ is not a Lie group, it is convenient: it reminds that G/H denotes the homogeneous space of positions and orientations.*

**Remark** **2.** *(notation and terminology)*

*Elements in Equation (3) denote equivalence classes of rigid body motions $g=\left(x,R_{n}\right)\in SE\left(3\right)$ that map (0,a) to (x,n):*

$$\left[g\right]=:\left(x,n\right)\in R^{3}⋊S^{2}\;\Leftrightarrow \;g⊙\left(0,a\right)=\left(x,n\right),$$

*under the (transitive) action*

(4) $$g⊙\left(x^{\prime },n^{\prime }\right)=\left(Rx^{\prime }+x,Rn^{\prime }\right),\;\text{for all}g=\left(x,R\right)\in SE\left(3\right),\;\left(x^{\prime },n^{\prime }\right)\in R^{3}⋊S^{2}.$$

*Therefore, we simply denote the equivalence classes [g] by (x,n). This is similar to the conventional writing $n\in S^{2}=SO\left(3\right)/SO\left(2\right)$. Throughout this manuscript, we refer to G/H as “the homogeneous space of positions and orientations” and henceforth $R_{n}$ denotes any rotation that maps the reference axis a into n.*

The precise definition of the Fourier transform $F_{G/H}$ on the homogeneous space G/H is presented in Section 4. It relies on the decomposition into unitary irreducible representations in Equation (2), but we must take both a domain and a range restriction into account. This is explained in Section 4. Next, we address an a priori domain constraint that is rather convenient than necessary.

**Remark** **3.** *We constrain the Fourier transform $F_{G/H}$ to*

(5) $$L_{2}^{sym}\left(G/H\right):=\left\{f\in L_{2}\left(G/H\right)\;|\;\forall_{R\in {\mathrm{Stab}}_{SO(3)}\left(a\right)}:\;f\left(x,n\right)=f\left(Rx,Rn\right)\right\}.$$

This constraint is convenient in view of the PDEs of interest (and the symmetries of their kernels) that we formulate in the next subsection, and that solve via Fourier’s method in Section 5.

### 1.3. Introduction to the PDEs of Interest on the Quotient $R^{3}⋊S^{2}$

Our main objective is to use the Fourier transform $F_{G/H}$ to solve the following PDEs on $R^{3}⋊S^{2}$:

(6) $$\begin{array}{c} \left\{\begin{array}{cc} \frac{\partial }{\partial t}W_{\alpha }\left(x,n,t\right) & =Q_{\alpha }W_{\alpha }\left(x,n,t\right),\\ W_{\alpha }\left(x,n,0\right) & =U(x,n), \end{array}\right. \end{array}$$

where $\left(x,n\right)\in R^{3}⋊S^{2}$, t≥0, α∈(0,1] and the generator

(7) $$Q_{\alpha }:=-{\left(-Q\right)}^{\alpha }$$

is expressed via

$$Q=D_{11}{∥n\times \nabla_{R^{3}}∥}^{2}+D_{33}{\left(n\cdot \nabla_{R^{3}}\right)}^{2}+D_{44}\Delta_{n}^{S^{2}},$$

with $D_{33}>D_{11}\geq 0,D_{44}>0$, and with $\Delta_{n}^{S^{2}}$ the Laplace–Beltrami operator on $S^{2}\;=\;\left\{n\in R^{3}\;|\;∥n∥=1\right\}$.

Note that the generator *Q* is a self-adjoint unbounded operator with domain

$$D\left(Q\right):=H_{2}\left(R^{3}\right)⊗H_{2}\left(S^{2}\right),$$

where $H_{2}$ denotes the Sobolev space $W_{2}^{2}$.

The semigroup for α=1 is a strongly continuous semigroup on $L_{2}\left(R^{3}⋊S^{2}\right)$ with a closed generator, and by taking the fractional power of the generator one obtains another strongly continuous semigroup, as defined and explained in a more general setting in the work by Yosida ([66], ch:11). The fractional power is formally defined by

(8) $$Q_{\alpha }W=-{\left(-Q\right)}^{\alpha }W:=\frac{\sin\alpha \pi }{\pi }\int_{0}^{\infty }\lambda^{\alpha -1}{\left(Q-\lambda I\right)}^{-1}\left(-QW\right)d\lambda \mathrm{for}\mathrm{all}W\in D\left(Q\right).$$

In Section 1.6, we show that the common technical representation Equation (8) is not really needed for our setting. In fact, it is very easy to account for α∈(0,1] in the solutions; by a spectral decomposition, we only need to take fractional powers of certain eigenvalues in the Fourier domain. For the moment, the reader may focus on the case α=1, where the system in Equation (6) becomes an ordinary elliptic diffusion system which is hypo-elliptic (in the sense of Hörmander [79]) even in the degenerate case where $D_{11}=0$.

The PDEs in Equation (6) have our interest as they are Forward-Kolmogorov equations for α-stable Lévy processes on G/H. See Appendix A for a precise formulation of discrete and continuous stochastic processes. This generalizes previous works on such basic processes [61,64] with applications in financial mathematics [80] and computer vision [65,78,81,82], from Lie group $R^{3}$ to the Lie group quotient $R^{3}⋊S^{2}$.

**Figure 1:**
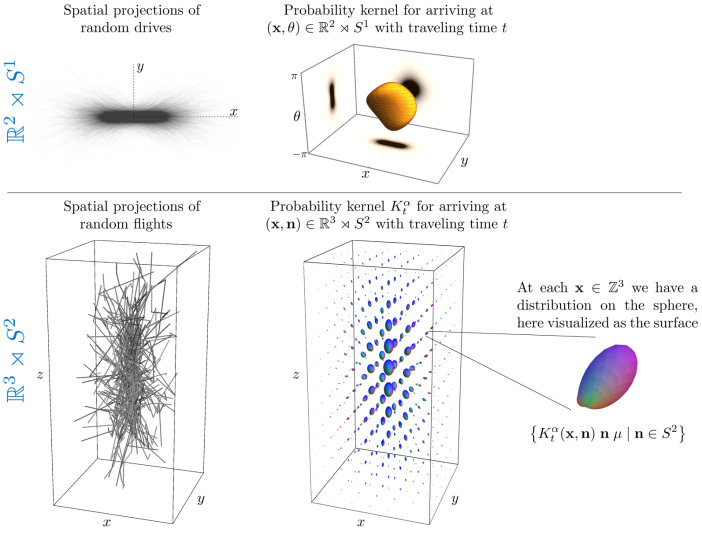
Various visualization of the diffusion process (α=1) on $R^{d}⋊S^{d-1}$, for d=2 and d=3. (**Top**) random walks (or rather “drunk man’s drives”) and an iso-contour of the limiting diffusion kernel, for the case d=2 studied in previous works (see, e.g., [15,37,83]); and (**Bottom**) random walks (or rather “drunk man’s flights”) and a visualization of the limiting distribution for the case d=3. This limiting distribution is a degenerate diffusion kernel $\left(x,n\right)\mapsto K_{t}^{\alpha =1}\left(x,n\right)$ that we study in this article. We visualize kernel $K_{t}^{\alpha =1}$ by a spatial grid of surfaces, where all surfaces are scaled by the same μ>0.

See Figure 1 for a visualization of sample paths from the discrete stochastic processes explained in Appendix A. They represent “drunk man’s flights” rather than “drunk man’s walks”.

### 1.4. Reformulation of the PDE on the Lie Group SE(3)

Now, we reformulate and extend our PDEs in Equation (6) to the Lie group G=SE(3) of rigid body motions, equipped with group product in Equation (1). This helps us to better recognize symmetries, as we show in Section 2.1. To this end, the PDEs are best expressed in a basis of left-invariant vector fields ${\left\{g\mapsto {\left.A_{i}\right|}_{g}\right\}}_{i=1}^{6}$ on *G*. Such left-invariant vector fields are obtained by push forward from the left-multiplication $L_{g_{1}}g_{2}:=g_{1}g_{2}$ as

$${\left.A_{i}\right|}_{g}={\left(L_{g}\right)}_{*}A_{i}\in T_{g}\left(G\right),$$

where $A_{i}:={\left.A_{i}\right|}_{e}$ form an orthonormal basis for the Lie algebra $T_{e}\left(G\right)$. We choose such a basis typically such that the first three are spatial generators $A_{1}=\partial_{x},A_{2}=\partial_{y},A_{3}=\partial_{z}=a\cdot \nabla_{R^{3}}$ and the remaining three are rotation generators, in such a way that $A_{6}$ is the generator of a counter-clockwise rotation around the reference axis a. For all $\tilde{U}\in C^{1}\left(G\right)$ and g∈G, one has

(9) $$A_{i}\tilde{U}\left(g\right)=\lim_{t↓0}\frac{\tilde{U}\left(g\;e^{tA_{i}}\right)-\tilde{U}\left(g\right)}{t},$$

where $A\mapsto e^{A}$ denotes the exponent that maps Lie algebra element $A\in T_{e}\left(G\right)$ to the corresponding Lie group element. The explicit formulas for the left-invariant vector fields in Euler-angles (requiring two charts) can be found in Appendix B, or in [4,84].

Now we can re-express the PDEs in Equation (6) on the group G=SE(3) as follows:

(10) $$\begin{array}{c} \left\{\begin{array}{ccc} \frac{\partial }{\partial t}{\tilde{W}}_{\alpha }\left(g,t\right) & ={\tilde{Q}}_{\alpha }{\tilde{W}}_{\alpha }\left(g,t\right), & g\in G,t\geq 0\\ {\tilde{W}}_{\alpha }\left(g,0\right) & =\tilde{U}\left(g\right), & g\in G, \end{array}\right. \end{array}$$

where the generator

(11) $${\tilde{Q}}_{\alpha }:=-{\left(-\tilde{Q}\right)}^{\alpha }$$

is again a fractional power (α∈(0,1]) of the diffusion generator $\tilde{Q}$ given by

(12) $$\tilde{Q}=D_{11}\left(A_{1}^{2}+A_{2}^{2}\right)+D_{33}\;A_{3}^{2}+D_{44}\left(A_{4}^{2}+A_{5}^{2}\right),$$

where $A_{i}^{2}=A_{i}\circ A_{i}$ for all i∈{1,…,5}. The initial condition in Equation (10) is given by

$$\tilde{U}\left(g\right)=\tilde{U}\left(x,R\right)=U\left(x,Ra\right).$$

Similar to the previous works [40,85], one has

(13) $$\begin{array}{c} {\tilde{W}}_{\alpha }\left(x,R,t\right)=W_{\alpha }\left(x,Ra,t\right), \end{array}$$

that holds for all t≥0,(x,R)∈SE(3).

**Remark** **4.** *Equation (13) relates the earlier PDE formulation in Equation (6) on the quotient G/H to the PDE formulation in Equation (10) on the group G. It holds since we have the relations*

$$\begin{array}{c} A_{6}{\tilde{W}}_{\alpha }\left(x,R,t\right)=0,\\ \left(A_{5}^{2}+A_{4}^{2}\right){\tilde{W}}_{\alpha }\left(x,R,t\right)=\Delta^{S^{2}}W_{\alpha }\left(x,Ra,t\right),\\ A_{3}{\tilde{W}}_{\alpha }\left(x,R_{n},t\right)=n\cdot \nabla_{R^{3}}W_{\alpha }\left(x,n,t\right),\\ \left(A_{1}^{2}+A_{2}^{2}\right){\tilde{W}}_{\alpha }\left(x,R,t\right)=\left(\Delta^{R^{3}}-A_{3}^{2}\right){\tilde{W}}_{\alpha }\left(x,R,t\right)={∥n\times \nabla_{R^{3}}∥}^{2}\;W_{\alpha }\left(x,Ra,t\right) \end{array}$$

*so that the generator of the PDE in Equation (10) on G and the generator of the PDE in Equation (6) on G/H indeed stay related via*

(14) $${\tilde{Q}}_{\alpha }{\tilde{W}}_{\alpha }\left(x,R,t\right)=Q_{\alpha }W_{\alpha }\left(x,Ra,t\right)\mathrm{for}\mathrm{all}t\geq 0.$$

### 1.5. Increase of Entropy for the Diffusion System (α=1) and the Poisson System ($\alpha =\frac{1}{2}$) on G/H

The PDE-system in Equation (6) on G/H relates to the PDE-system in Equation (10) on *G* via Equation (14). Next, we show that for $\alpha =\frac{1}{2}$ the PDE-system boils down to a Poisson system. For α=1 the PDE-system in Equation (10) is a diffusion system on Lie group *G*, for which one has monotonic increase of entropy [75]. The next theorem quantifies the monotonic increase of entropy for $\alpha \in \{\frac{1}{2},1\}$ in terms of Fisher matrices.

**Definition** **4.** *Let α∈(0,1]. Let ${\tilde{W}}_{\alpha }$ be the solution to Equation (10) with positive initial condition $\tilde{U}>0$ with $\tilde{U}\in L_{2}\left(G\right)$ and $\int_{G}\tilde{U}\left(g\right)dg=1$. Then, we define the entropy $E_{\alpha }\left(t\right)$ at evolution time t≥0 as*

(15) $$E_{\alpha }\left(t\right):=-\int_{G}{\tilde{W}}_{\alpha }\left(g,t\right)\;\log\;{\tilde{W}}_{\alpha }\left(g,t\right)\;dg.$$

**Proposition** **1.** *For $\alpha =\frac{1}{2}$, the PDE system in (10) yields the same solutions as the following Poisson system:*

(16) $$\left\{\begin{array}{cccc} \left(\frac{\partial^{2}}{\partial t^{2}}+\tilde{Q}\right){\tilde{W}}_{\frac{1}{2}}\left(g,t\right) & = & 0 & \;g\in G,t\geq 0,\;\mathrm{with}\forall_{t\geq 0}:{\tilde{W}}_{\frac{1}{2}}\left(\cdot ,t\right)\in L_{2}\left(G\right)\\ {\tilde{W}}_{\frac{1}{2}}\left(g,0\right) & = & \tilde{U}\left(g\right)>0, & \;g\in G. \end{array}\right.$$

*The entropy in Equation (15) equals $E_{\alpha }\left(t\right)=-2\pi \int_{G/H}W_{\alpha }\left(x,n,t\right)\;\log\;W_{\alpha }\left(x,n,t\right)\;dxd\mu_{S^{2}}\left(n\right)$.*

*For all t>0, one has*

(17) $$\begin{array}{c} E_{1}^{\prime }\left(t\right)=\mathrm{trace}\left\{D\cdot F_{1}\left(t\right)\right\}>0,\\ E_{\frac{1}{2}}^{\prime \prime }\left(t\right)<-\mathrm{trace}\left\{D\cdot F_{\frac{1}{2}}\left(t\right)\right\}<0\mathrm{and}E_{\frac{1}{2}}^{\prime }\left(t\right)=\int_{t}^{\infty }\mathrm{trace}\left\{D\cdot F_{\frac{1}{2}}\left(\tau \right)\right\}+F\left(\tau \right)\;d\tau >0, \end{array}$$

*for the diffusion matrix $D=\mathrm{diag}{\left\{D_{ii}\right\}}_{i=1}^{6}>0$, where $D_{11}=D_{22}$, $D_{33}$ and $D_{44}=D_{55}$ are the coefficients in $\tilde{Q}$, and with Fisher matrix $F_{\alpha }\left(t\right)=\mathrm{diag}{\left\{\int_{G}\frac{{\left|A_{i}{\tilde{W}}_{\alpha }\left(g,t\right)\right|}^{2}}{{\tilde{W}}_{\alpha }\left(g,t\right)}\;dg\right\}}_{i=1}^{6}$, and $F\left(t\right)=\int_{G}\frac{|\partial_{\tau }{\tilde{W}}_{1/2}{\left(g,t\right)|}^{2}}{{\tilde{W}}_{1/2}\left(g,t\right)}dg\geq 0$.*

**Proof.**  For $\alpha =\frac{1}{2}$, one has by the square integrability constraint in Equation (16) and application of the unitary Fourier transform on *G* that $\left(\frac{\partial^{2}}{\partial t^{2}}+\tilde{Q}\right){\tilde{W}}_{\frac{1}{2}}=\left(\frac{\partial }{\partial t}-\sqrt{-\tilde{Q}}\right)\left(\frac{\partial }{\partial t}+\sqrt{-\tilde{Q}}\right){\tilde{W}}_{\frac{1}{2}}=0\Rightarrow \left(\frac{\partial }{\partial t}+\sqrt{-\tilde{Q}}\right){\tilde{W}}_{\frac{1}{2}}=0$ and thereby the PDE system in Equation (10) can be replaced by the Poisson system in Equation (16) on $G\times R^{+}$. The formula for the entropy follows from a product decomposition of the (bi-invariant) haar measure on *G* into measure on the quotient G/H and a measure on the subgroup H≡{0}×SO(2) and the fact that ${\tilde{W}}_{\alpha }\left(gh,t\right)={\tilde{W}}_{\alpha }\left(g,t\right)$ for all h∈H, α∈(0,1], due to Equation (14). For $\alpha =\frac{1}{2}$, we have that ${\tilde{W}}_{\alpha }$ satisfies Equation (16) and

$$\begin{array}{cc} E_{\frac{1}{2}}^{\prime \prime }\left(t\right) & =-\int_{G}\frac{{\left(\partial_{t}{\tilde{W}}_{\frac{1}{2}}\left(g,t\right)\right)}^{2}}{{\tilde{W}}_{\frac{1}{2}}\left(g,t\right)}-\left(\log\left({\tilde{W}}_{\frac{1}{2}}\left(g,t\right)+1\right)\right)\;\partial_{t}^{2}{\tilde{W}}_{\frac{1}{2}}\left(g,t\right)\;dg\\ & <\int_{G}\left(\log{\tilde{W}}_{\frac{1}{2}}\left(g,t\right)+1\right)\;\tilde{Q}{\tilde{W}}_{\frac{1}{2}}\left(g,t\right)\;dg=\int_{G}\left(\log{\tilde{W}}_{\frac{1}{2}}\left(g,t\right)\right)\;\tilde{Q}{\tilde{W}}_{\frac{1}{2}}\left(g,t\right)\;dg\\ & =-\int_{G}\sum_{i=1}^{6}\frac{D_{ii}{\left|A_{i}{\tilde{W}}_{\frac{1}{2}}\left(g,t\right)\right|}^{2}}{{\tilde{W}}_{\frac{1}{2}}\left(g,t\right)}\;dg=-\mathrm{trace}\left(D\cdot F_{\frac{1}{2}}\left(t\right)\right), \end{array}$$

where we use integration by parts and short notation with $\partial_{t}:=\frac{\partial }{\partial t}$. Now, $E_{\frac{1}{2}}^{\prime \prime }<0$ and $E_{\frac{1}{2}}^{\prime }$ is continuous (due to the Lebesgue dominated convergence principle and continuity of each mapping $t\mapsto \partial_{t}\tilde{W}\left(g,t\right)$ indexed by g∈G) and $E_{\frac{1}{2}}\left(t\right)\to 0$ when t→∞, from which we deduce that $E_{\frac{1}{2}}^{\prime }\left(t\right)=-\;\int_{t}^{\infty }E_{\frac{1}{2}}^{\prime \prime }\left(\tau \right)d\tau >0$.

For α=1, we follow ([75], Thm.2) and compute (again using the PDE and integration by parts)

$$E_{1}^{\prime }\left(t\right)=-\;\int_{G}\left(\partial_{t}{\tilde{W}}_{1}\left(g,t\right)\right)\log{\tilde{W}}_{1}\left(g,t\right)+{\tilde{W}}_{1}\left(g,t\right)\;dg=\int_{G}\sum_{i=1}^{6}D_{ii}\frac{|A_{i}{\tilde{W}}_{1}{\left(g,t\right)|}^{2}}{{\tilde{W}}_{1}\left(g,t\right)}\;dg=\mathrm{trace}\left(D\cdot F_{1}\left(t\right)\right)>0.$$

Regarding the strict positivity in Equation (17), we note that $\tilde{U}>0\Rightarrow {\tilde{W}}_{\alpha }>0$ and if $E_{\alpha }^{\prime }\left(t\right)=0$ then this would imply that ${\tilde{W}}_{\alpha }\left(\cdot ,t\right)$ is constant, which violates ${\tilde{W}}_{\alpha }\left(\cdot ,t\right)\in L_{2}\left(G\right)$ as *G* is not compact. □

### 1.6. A Preview on the Spectral Decomposition of the PDE Evolution Operator and the Inclusion of α

Let *U* be in the domain of the generator $Q_{\alpha }$ given by Equation (7), of our evolution Equation (6). For a formal definition of this domain, we refer to ([86], Equation 9). Let its spatial Fourier transform be given by

(18) $$\bar{U}\left(\omega ,n\right)=\left[F_{R^{3}}U\left(\cdot ,n\right)\right]\left(\omega \right):=\frac{1}{{\left(2\pi \right)}^{\frac{3}{2}}}\int_{R^{3}}U\left(x,n\right)\;e^{-i\omega \cdot x}\;dx.$$

To the operator $Q_{\alpha }$, we associate the corresponding operator $-{\left(-B\right)}^{\alpha }$ in the spatial Fourier domain by

(19) $$-{\left(-B\right)}^{\alpha }=\left(F_{R^{3}}⊗1_{L_{2}\left(S^{2}\right)}\right)\circ Q_{\alpha }\circ \left(F_{R^{3}}^{-1}⊗1_{H_{2\alpha }\left(S^{2}\right)}\right).$$

Then, direct computations show us that

(20) $$-{\left(-B\right)}^{\alpha }\bar{U}\left(\omega ,n\right)=\left[-{\left(-B_{\omega }\right)}^{\alpha }\bar{U}\left(\omega ,\cdot \right)\right]\left(n\right),\mathrm{for}\mathrm{all}n\in S^{2},$$

where, for each fixed $\omega \in R^{3}$, the operator $-{\left(-B_{\omega }\right)}^{\alpha }:H_{2\alpha }\left(S^{2}\right)\to L_{2}\left(S^{2}\right)$ is given by

(21) $$-{\left(-B_{\omega }\right)}^{\alpha }=-{\left(-D_{44}\Delta_{n}^{S^{2}}+D_{11}{∥\omega \times n∥}^{2}+D_{33}{\left(\omega \cdot n\right)}^{2}\right)}^{\alpha }.$$

In this article, we employ Fourier transform techniques to derive a complete orthonormal basis (ONB) of eigenfunctions

(22) $$\left\{\Phi_{\omega }^{l,m}\;|\;l\in N_{0},m\in Z\mathrm{with}\left|m\right|\leq l\right\},$$

in $L_{2}\left(S^{2}\right)$ for the operator $-\left(-B_{\omega }\right):=-{\left(-B_{\omega }\right)}^{\alpha =1}$. Then, clearly, this basis is also an ONB of eigenfunctions for $-{\left(-B_{\omega }\right)}^{\alpha }$, as we only need to take the fractional power of the eigenvalues. Indeed, once the eigenfunctions in Equation (22) and the eigenvalues

(23) $$B_{\omega }\Phi_{\omega }^{l,m}=\lambda_{r}^{l,m}\;\Phi_{\omega }^{l,m},\mathrm{with}r=∥\omega ∥,$$

are known, the exact solution of Equation (6) is given by (shift-twist) convolution with a probability kernel on $R^{3}⋊S^{2}$. More precisely, the solutions of Equation (6) can be expressed as follows:

(24) $$\begin{array}{c} W_{\alpha }\left(x,n,t\right)=\left(K_{t}^{\alpha }*U\right)\left(x,n\right):=\int_{S^{2}}\int_{R^{3}}K_{t}^{\alpha }\left(R_{n^{\prime }}^{T}\left(x-x^{\prime }\right),R_{n^{\prime }}^{T}n\right)\;U\left(x^{\prime },n^{\prime }\right)\;dx^{\prime }d\mu_{S^{2}}\left(n^{\prime }\right)\\ =\int_{R^{3}}\sum_{l=0}^{\infty }\sum_{m=-l}^{l}\;{\langle \;\bar{U}\left(\omega ,\cdot \right)\;,\;\Phi_{\omega }^{l,m}\left(\cdot \right)\;\rangle }_{L_{2}\left(S^{2}\right)}\Phi_{\omega }^{l,m}\left(n\right)\;e^{-{\left(-\lambda_{r}^{l,m}\right)}^{\alpha }t}e^{ix\cdot \omega }d\omega ,\\ \mathrm{with}\mathrm{the}\mathrm{probability}\mathrm{kernel}\mathrm{given}\mathrm{by}\\ \;K_{t}^{\alpha }\left(x,n\right)=\left[F_{R^{3}}^{-1}\left({\bar{K}}_{t}^{\alpha }\left(\cdot ,n\right)\right)\right]\left(x\right),\\ \;\;\;\mathrm{with}{\bar{K}}_{t}^{\alpha }\left(\omega ,n\right)=\sum_{l=0}^{\infty }\sum_{m=-l}^{l}\bar{\Phi_{\omega }^{l,m}\left(a\right)}\;\Phi_{\omega }^{l,m}\left(n\right)\;e^{-{\left(-\lambda_{r}^{l,m}\right)}^{\alpha }t}. \end{array}$$

Here, the inner product in $L_{2}\left(S^{2}\right)$ is given by

(25) $${\langle y_{1}\left(\cdot \right),y_{2}\left(\cdot \right)\rangle }_{L_{2}\left(S^{2}\right)}:=\int_{S^{2}}y_{1}\left(n\right)\;\bar{y_{2}\left(n\right)}\;d\mu_{S^{2}}\left(n\right).$$

where $\mu_{S^{2}}$ is the usual Lebesgue measure on the sphere $S^{2}$.

**Remark** **5.** *The eigenvalues $\lambda_{r}^{l,m}$ only depend on r=∥ω∥ due to the symmetry $\Phi_{R\omega }^{l,m}\left(Rn\right)=\Phi_{\omega }^{l,m}\left(n\right)$ that one directly recognizes from Equations (21) and (23).*

**Remark** **6.** *The kernels $K_{t}^{\alpha }$ are the probability density kernels of stable Lévy processes on $R^{3}⋊S^{2}$, see Appendix A.1. Therefore, akin to the $R^{n}$-case [61,65], we refer to them as the α-stable Lévy kernels on $R^{3}⋊S^{2}$.*

## 2. Symmetries of the PDEs of Interest

Next, we employ the PDE formulation in Equation (10) on the group G=SE(3) to summarize the symmetries for the probability kernels $K_{t}^{\alpha }:R^{3}⋊S^{2}\to R^{+}$. For details, see [40,87].

### 2.1. PDE Symmetries

Consider the PDE system in Equation (10) on the group G=SE(3). Due to left-invariance (or rather left-covariance) of the PDE, linearity of the map $\tilde{U}\left(\cdot \right)\mapsto {\tilde{W}}_{\alpha }\left(\cdot ,t\right)$, and the Dunford–Pettis theorem [88], the solutions are obtained by group convolution with a kernel ${\tilde{K}}_{t}^{\alpha }\in L_{1}\left(G\right)$:

(26) $${\tilde{W}}_{\alpha }\left(g,t\right)=\left({\tilde{K}}_{t}^{\alpha }*\tilde{U}\right)\left(g\right):=\int_{G}{\tilde{K}}_{t}^{\alpha }\left(h^{-1}g\right)\;\tilde{U}\left(h\right)\;dh,$$

where we take the convention that the probability kernel acts from the left. In the special case, $U=\delta_{e}$ with unity element e=(0,I) we get ${\tilde{W}}_{\alpha }\left(g,t\right)={\tilde{K}}_{t}^{\alpha }\left(g\right)$.

Thanks to the fundamental relation in Equation (13) that holds in general, we have in particular that

(27) $$\begin{array}{c} \forall_{t\geq 0}\;\forall_{(x,R)\in G}\;:\;{\tilde{K}}_{t}^{\alpha }\left(x,R\right)=K_{t}^{\alpha }\left(x,Ra\right). \end{array}$$

Furthermore, the PDE system given by Equation (10) is invariant under $A_{i}\mapsto -A_{i}$, and, since inversion on the Lie algebra corresponds to inversion on the group, the kernels must satisfy

(28) $$\forall_{t\geq 0}\;\forall_{g\in G}\;:\;{\tilde{K}}_{t}^{\alpha }\left(g\right)={\tilde{K}}_{t}^{\alpha }\left(g^{-1}\right),$$

and for the corresponding kernel on the quotient this means

(29) $$\forall_{t\geq 0}\;\forall_{(x,n)\in G/H}\;:\;K_{t}^{\alpha }\left(x,n\right)=K_{t}^{\alpha }\left(-R_{n}^{T}x,R_{n}^{T}a\right).$$

Finally, we see invariance of the PDE with respect to right actions of the subgroup *H*. This is due to the isotropy of the generator ${\tilde{Q}}_{\alpha }$ in the tangent subbundles $\mathrm{span}\{A_{1},A_{2}\}$ and $\mathrm{span}\{A_{4},A_{5}\}$. This due to Equation (A11) in Appendix B. Note that invariance of the kernel with respect to right action of the subgroup *H* and invariance of the kernel with respect to inversion in Equation (28) also implies invariance of the kernel with respect to left-actions of the subgroup *H*, since ${\left(g^{-1}{\left(h^{\prime }\right)}^{-1}\right)}^{-1}=h^{\prime }g$ for all $h^{\prime }\in H$ and g∈G. Therefore, we have

(30) $$\begin{array}{ccc} \forall_{t\geq 0}\;\forall_{g\in G}\forall_{h,h^{\prime }\in H}:\; & {\tilde{K}}_{t}^{\alpha }\left(\;g\;h\;\right) & ={\tilde{K}}_{t}^{\alpha }\left(g\right)={\tilde{K}}_{t}^{\alpha }\left(h^{\prime }g\right),\\ \forall_{t\geq 0}\;\forall_{(x,n)\in G/H}\forall_{\bar{\alpha }\in \left[0,2\pi \right)}:\; & K_{t}^{\alpha }\left(x,n\right) & =K_{t}^{\alpha }\left(R_{a,\bar{\alpha }}x,R_{a,\bar{\alpha }}n\right). \end{array}$$

**Remark** **7.** *(notations, see also the list of abbreviations at the end of the article)*

*To avoid confusion between the Euler angle $\bar{\alpha }$ and the α indexing the α-stable Lévy distribution, we put an overline for this specific angle. Henceforth, $R_{v,\psi }$ denotes a counter-clockwise rotation over axis v with angle ψ. This applies in particular to the case where the axis is the reference axis $v=a={\left(0,0,1\right)}^{T}$ and $\psi =\bar{\alpha }$. Recall that $R_{n}$ (without an angle in the subscript) denotes any 3D rotation that maps reference axis a onto n.*

*We write the symbol $\hat{\left.\cdot \right.}$ above a function to indicate its Fourier transform on G and G/H; we use the symbol $\bar{\left.\cdot \right.}$ for strictly spatial Fourier transform; the symbol $\tilde{\left.\cdot \right.}$ above a function/operator to indicate that it is defined on the group G and the function/operator without symbols when it is defined on the quotient G/H.*

### 2.2. Obtaining the Kernels with $D_{11}>0$ from the Kernels with $D_{11}=0$

In ([40], cor.2.5), it was deduced that for α=1 the elliptic diffusion kernel ($D_{11}>0$) directly follows from the degenerate diffusion kernel ($D_{11}=0$) in the spatial Fourier domain via

$${\bar{K}}_{t}^{1,\mathrm{elliptic}}\left(\omega ,n\right)=e^{-r^{2}D_{11}t}{\bar{K}}_{t}^{1,\mathrm{degenerate}}\left(\sqrt{\frac{D_{33}-D_{11}}{D_{33}}}\omega ,n\right),\;\mathrm{with}\;r=∥\omega ∥,\;\;0\leq D_{11}<D_{33}.$$

For the general case α∈(0,1], the transformation from the case $D_{11}=0$ to the case $D_{11}>0$ is achieved by replacing $-{\left(-\lambda_{r}^{l,m}\right)}^{\alpha }\mapsto -{\left(-\lambda_{r}^{l,m}+r^{2}D_{11}\right)}^{\alpha }\mathrm{and}$ $r\mapsto r\sqrt{\frac{D_{33}-D_{11}}{D_{33}}}$ in Equation (24) for the kernel. Henceforth, we set $D_{11}=0$.

## 3. The Fourier Transform on SE(3)

The group G=SE(3) is a unimodular Lie group (of type I) with (left- and right-invariant) Haar measure $dg=dxd\mu_{SO(3)}\left(R\right)$ being the product of the Lebesgue measure on $R^{3}$ and the Haar measure $\mu_{SO(3)}$ on SO(3). Then, for all $f\in L_{1}\left(G\right)\cap L_{2}\left(G\right)$, the Fourier transform $F_{G}f$ is given by Equation (2). For more detailsm see [22,24,26]. One has the inversion formula:

(31) $$f\left(g\right)=\left(F_{G}^{-1}F_{G}f\right)\left(g\right)=\int_{\hat{G}}\mathrm{trace}\left\{\left(F_{G}f\right)\left(\sigma \right)\;\sigma_{g}\right\}d\nu \left(\sigma \right)=\int_{\hat{G}}\mathrm{trace}\left\{\hat{f}\left(\sigma \right)\;\sigma_{g}\right\}d\nu \left(\sigma \right).$$

In our Lie group case of SE(3), we identify all unitary irreducible representations $\sigma^{p,s}$ having non-zero dual measure with the pair $\left(p,s\right)\in R^{+}\times Z$. This identification is commonly applied (see, e.g., [4]). Using the method ([26], Thm. 2.1, [25]) of induced representations, all unitary irreducible representations (UIRs) of *G*, up to equivalence, with non-zero Plancherel measure are given by:

(32) $$\begin{array}{c} \begin{array}{c} \sigma =\sigma^{p,s}:SE\left(3\right)\to B\left(L_{2}\left(p\;S^{2}\right)\right),\;p>0,\;s\in Z,\\ \left(\sigma_{\left(x,R\right)}^{p,s}\phi \right)\left(u\right)=e^{-i\;u\cdot x}\;\phi \left(R^{-1}u\right)\;\Delta_{s}\left(R_{\frac{u}{p}}^{-1}RR_{\frac{R^{-1}u}{p}}\right),\;\;\;u\in pS^{2},\;\phi \in L_{2}\left(pS^{2}\right), \end{array} \end{array}$$

where $pS^{2}$ denotes a 2D sphere of radius p=∥u∥; $\Delta_{s}$ is a unitary irreducible representation of SO(2) (or rather of the stabilizing subgroup ${\mathrm{Stab}}_{SO(3)}\left(a\right)\subset SO\left(3\right)$ isomorphic to SO(2)) producing a scalar.

In Equation (32), $R_{\frac{u}{p}}$ denotes a rotation that maps a onto $\frac{u}{p}$. Thus, direct computation

$$R_{\frac{u}{p}}^{-1}RR_{\frac{R^{-1}u}{p}}a=R_{\frac{u}{p}}^{-1}RR^{-1}\left(\frac{u}{p}\right)=a$$

shows us that it is a rotation around the *z*-axis (recall $a=e_{z}$), e.g., about angle $\bar{\alpha }$. This yields character $\Delta_{s}\left(R_{\frac{u}{p}}^{-1}RR_{\frac{R^{-1}u}{p}}\right)=e^{-is\bar{\alpha }}$, for details, see ([4], ch.10.6). Thus, we can rewrite Equation (32) as

$$\left(\sigma_{\left(x,R\right)}^{p,s}\phi \right)\left(u\right)=e^{-i\;\left(u\cdot x+s\bar{\alpha }\right)}\;\phi \left(R^{-1}u\right),\;\;\mathrm{where}\left(x,R\right)\in G,\;u\in pS^{2},\;\phi \in L_{2}\left(pS^{2}\right).$$

Mackey’s theory [25] relates the UIR $\sigma^{p,s}$ to the dual orbits $pS^{2}$ of SO(3). Thereby, the dual measure ν can be identified with a measure on the family of dual orbits of SO(3) given by $\left\{pS^{2}\;|\;p>0\right\}$, and

$$\left(F_{G}^{-1}\hat{f}\right)\left(g\right)=\int_{\hat{G}}\mathrm{trace}\left\{\hat{f}\left(\sigma^{p,s}\right)\;\sigma_{g}^{p,s}\right\}\;d\nu \left(\sigma^{p,s}\right)=\int_{R^{+}}\mathrm{trace}\left\{\hat{f}\left(\sigma^{p,s}\right)\;\sigma_{g}^{p,s}\right\}p^{2}dp,$$

for all p>0, s∈Z. For details, see ([24], ch. 3.6.).

The matrix elements of $\hat{f}=F_{G}f$ with respect to an orthonormal basis of modified spherical harmonics $\left\{Y_{s}^{l,m}\left(p^{-1}\cdot \right)\right\}$, with |m|,|s|≤l (see ([4], ch.9.8)) for $L_{2}\left(pS^{2}\right)$ are given by

(33) $${\hat{f}}_{l,m,l^{\prime },m^{\prime }}^{p,s}:=\int_{G}f\left(g\right)\;{\langle \;\sigma_{g^{-1}}^{p,s}Y_{s}^{l^{\prime },m^{\prime }}\left(p^{-1}\;\cdot \right)\;,\;Y_{s}^{l,m}\left(p^{-1}\;\cdot \right)\rangle }_{L_{2}\left(pS^{2}\right)}\;\;dg,$$

where the $L_{2}$ inner product is given by ${\langle \;y_{1}\left(\cdot \right)\;,\;y_{2}\left(\cdot \right)\;\rangle }_{L_{2}\left(pS^{2}\right)}:={\langle \;y_{1}\left(p\cdot \right)\;,\;y_{2}\left(p\cdot \right)\;\rangle }_{L_{2}\left(S^{2}\right)}$ (recall Equation (25)).

For an explicit formula for the modified spherical harmonics $Y_{s}^{l,m}$ see [4], where they are denoted by $h_{m,s}^{l}$. The precise technical analytic expansion of the modified spherical harmonics is not important for this article. The only properties of $Y_{s}^{l,m}$ that we need are gathered in the next proposition.

**Proposition** **2.** *The modified spherical harmonics $Y_{s}^{l,m}$ have the following properties:*

*(1)*  *For s = *0* or m = *0*, they coincide with standard spherical harmonics Y^l,m^, cf. ([89], eq.4.32):*
  $$\begin{array}{c} \;\;Y_{s=0}^{l,m}=Y^{l,m}\mathrm{and}Y_{s}^{l,0}={\left(-1\right)}^{s}\;Y^{l,s},\mathrm{where}Y^{l,m}\left(n\left(\beta ,\gamma \right)\right)=\frac{\epsilon_{m}}{\sqrt{2\pi }}\;P_{l}^{m}\left(\cos\beta \right)\;e^{im\gamma },\\ \mathrm{with}n\left(\beta ,\gamma \right)={\left(\cos\gamma \sin\beta ,\sin\gamma \sin\beta ,\cos\beta \right)}^{T},\mathrm{with}\mathrm{spherical}\mathrm{angles}\beta \in \left[0,\pi \right],\gamma \in \left[0,2\pi \right),\\ \mathrm{with}P_{l}^{m}\text{the normalized associated Legendre polynomial and }\epsilon_{m}={\left(-1\right)}^{\frac{1}{2}\left(m+|m|\right)}. \end{array}$$
*(2)*  *They have a specific rotation transformation property in view of Equation (32):*
  $$\begin{array}{c} \;\;\sigma_{\left(0,R\right)}^{p,s}Y_{s}^{l,m}=\sum_{m^{\prime }=-l}^{l}D_{m^{\prime }m}^{l}\left(R\right)\;Y_{s}^{l,m^{\prime }},\mathrm{where}D_{m^{\prime }m}^{l}\left(\cdot \right)\mathrm{denotes}\mathrm{the}\mathrm{Wigner}D-\mathrm{function}\;\mathrm{Wigner}. \end{array}$$
*(3)*  *For each s* ∈ Z *fixed, they form a complete orthonormal basis for* L^2^(*S*^2^)*:*
  $$\begin{array}{c} \;{\langle Y_{s}^{l,m}\left(\cdot \right),Y_{s}^{l^{\prime },m^{\prime }}\left(\cdot \right)\rangle }_{L_{2}\left(S^{2}\right)}=\delta^{l,l^{\prime }}\delta^{m,m^{\prime }}\mathrm{for}\mathrm{all}m,m^{\prime }\in Z,\;l,l^{\prime }\in N_{0},\;\mathrm{with}|m|\leq l,|m^{\prime }|\leq l^{\prime },\;l,l^{\prime }\geq \left|s\right|. \end{array}$$

For details and relation between different Euler angle conventions, see ([4], ch:9.4.1). In our convention of ZYZ-Euler angles (see Appendix B), one has

(34) $$D_{m^{\prime }m}^{l}\left(R_{e_{z},\bar{\alpha }}R_{e_{y},\beta }R_{e_{z},\gamma }\right)=e^{-im^{\prime }\bar{\alpha }}P_{m^{\prime }m}^{l}\left(\cos\beta \right)e^{-im\gamma },$$

with $P_{m^{\prime }m}^{l}$ a generalized associated Legendre polynomial given in ([4], eq.9.21). insertremarks

Moreover, we have inversion formula ([4], Equation 10.46):

(35) $$f\left(g\right)=\frac{1}{2\pi^{2}}\sum_{s\in Z}\sum_{l^{\prime }=\left|s\right|}^{\infty }\sum_{l=|s|}^{\infty }\sum_{m^{\prime }=-l^{\prime }}^{l^{\prime }}\sum_{m=-l}^{l}\int_{0}^{\infty }{\hat{f}}_{l,m,l^{\prime },m^{\prime }}^{p,s}\;{\left(\sigma_{g}^{p,s}\right)}_{l^{\prime },m^{\prime },l,m}\;p^{2}dp,$$

with matrix coefficients (independent of *f*) given by

(36) $${\left(\sigma_{g}^{p,s}\right)}_{l^{\prime },m^{\prime },l,m}={\langle \;\sigma_{g}^{p,s}Y_{s}^{l,m}\left(p^{-1}\cdot \right)\;,\;Y_{s}^{l^{\prime },m^{\prime }}\left(p^{-1}\cdot \right)\;\rangle }_{L_{2}\left(pS^{2}\right)}.$$

Note that $\sigma^{p,s}$ is a UIR so we have

(37) $${\left(\sigma_{g^{-1}}^{p,s}\right)}_{l^{\prime },m^{\prime },l,m}=\bar{{\left(\sigma_{g}^{p,s}\right)}_{l,m,l^{\prime },m^{\prime }}}\;.$$

## 4. A Specific Fourier Transform on the Homogeneous Space $R^{3}⋊S^{2}$ of Positions and Orientations

Now that we have introduced the notation of Fourier transform on the Lie group G=SE(3), we define the Fourier transform $F_{G/H}$ on the homogeneous space $G/H=R^{3}⋊S^{2}$. Afterwards, in the subsequent section, we solve the Forward-Kolmogorov/Fokker–Planck PDEs in Equation (6) via application of this transform, or, more precisely, via conjugation with Fourier transform $F_{G/H}$.

### 4.1. The Homogeneous Space $R^{3}⋊S^{2}$

Throughout this manuscript, we rely on a Fourier transform on the homogeneous space of positions and orientations that is defined by the partition of left-cosets: $R^{3}⋊S^{2}:=G/H$, given by Equation (3).

Note that subgroup *H* can be parameterized as follows:

(38) $$H=\{h_{\bar{\alpha }}:=\left(0,R_{a,\bar{\alpha }}\right)\;|\;\bar{\alpha }\in \left[0,2\pi \right)\},$$

where we recall that $R_{a,\bar{\alpha }}$ denotes a (counter-clockwise) rotation around the reference axis $a=e_{z}$. The reason behind this construction is that the group SE(3) acts transitively on $R^{3}⋊S^{2}$ by $\left(x^{\prime },n^{\prime }\right)\mapsto g⊙\left(x^{\prime },n^{\prime }\right)$ given by Equation (4). Recall that by the definition of the left-cosets one has

$$H=\left\{0\right\}\times SO\left(2\right),\mathrm{and}g_{1}∼g_{2}\Leftrightarrow g_{1}^{-1}g_{2}\in H.$$

The latter equivalence simply means that for $g_{1}=\left(x_{1},R_{1}\right)$ and $g_{2}=\left(x_{2},R_{2}\right)$ one has

$$g_{1}∼g_{2}\Leftrightarrow x_{1}=x_{2}\mathrm{and}\exists_{\bar{\alpha }\in \left[0,2\pi \right)}\;:\;R_{1}=R_{2}R_{a,\bar{\alpha }}.$$

The equivalence classes $\left[g\right]=\{g^{\prime }\in SE\left(3\right)\;|\;g^{\prime }∼g\}$ are often just denoted by (x,n) as they consist of all rigid body motions $g=(x,R_{n})$ that map reference point (0,a) onto $\left(x,n\right)\in R^{3}⋊S^{2}$:

(39) g⊙(0,a)=(x,n),

where we recall $R_{n}$ is *any* rotation that maps $a\in S^{2}$ onto $n\in S^{2}$.

### 4.2. Fourier Transform on $R^{3}⋊S^{2}$

Now we can define the Fourier transform $F_{G/H}$ on the homogeneous space G/H. Prior to this, we specify a class of functions where this transform acts.

**Definition** **5.** *Let p>0 be fixed and s∈Z. We denote*

$$L_{2}^{sym}\left(pS^{2}\right)=\left\{\left.f\in L_{2}\left(pS^{2}\right)\;\right|\;\forall_{\bar{\alpha }\in \left[0,2\pi \right)}\;\sigma_{h_{\bar{\alpha }}}^{p,s}f=f\right\}$$

*the subspace of spherical functions that have the prescribed axial symmetry, with respect to the subgroup H (recall Equation (38)).*

**Definition** **6.** *We denote the orthogonal projection from $L_{2}\left(pS^{2}\right)$ onto the closed subspace $L_{2}^{sym}\left(pS^{2}\right)$ by $P_{p}^{sym}$.*

**Definition** **7.** *To the group representation $\sigma^{p,s}:SE\left(3\right)\to B\left(L_{2}\left(pS^{2}\right)\right)$ given by Equation (32), we relate a “representation” ${\bar{\sigma }}^{p,s}:R^{3}⋊S^{2}\to B\left(L_{2}\left(pS^{2}\right)\right)$ on $R^{3}⋊S^{2}$, defined by*

(40) $${\bar{\sigma }}_{\left[g\right]}^{p,s}:=\frac{1}{{\left(2\pi \right)}^{2}}\int_{0}^{2\pi }\int_{0}^{2\pi }\sigma_{h_{\tilde{\alpha }}gh_{\bar{\alpha }}}^{p,s}\;d\bar{\alpha }d\tilde{\alpha }=P_{p}^{sym}\circ \sigma_{g}^{p,s}\circ P_{p}^{sym}.$$

**Definition** **8.** *A function $\tilde{U}:G\to C$ is called axially symmetric if*

(41) $$\tilde{U}\left(x,R\right)=\tilde{U}\left(x,RR_{a,\bar{\alpha }}\right)\;\;\;\mathrm{for}\mathrm{all}\bar{\alpha }\in \left[0,2\pi \right)\mathrm{and}\mathrm{all}\left(x,R\right)\in G.$$

To each function U:G/H→C, we relate an axially symmetric function $\tilde{U}:G\to C$ by

(42) $$\tilde{U}\left(x,R\right):=U\left(x,Ra\right).$$

**Definition** **9.** *We define the Fourier transform of function U on $G/H=R^{3}⋊S^{2}$ by*

(43) $$\begin{array}{c} \hat{U}\left({\bar{\sigma }}^{p,s}\right)=\left(F_{G/H}U\right)\left({\bar{\sigma }}^{p,s}\right):=P_{p}^{sym}\circ F_{G}\tilde{U}\left(\sigma^{p,s}\right)\circ P_{p}^{sym}. \end{array}$$

Standard properties of the Fourier transform $F_{G}$ on SE(3) such as the Plancherel theorem and the inversion formula [4,26] naturally carry over to $F_{G/H}$ with “simpler formulas”. This is done by a domain and range restriction via the projection operators $P_{p}^{sym}$ in Equation (43). The reason for the specific construction Equation (43) becomes clear from the next lemmas, and the “simpler formulas” for the Plancherel and inversion formulas are then summarized in a subsequent theorem, where we constrain ourselves to the case $m=m^{\prime }=0$ in the formulas. The operator $P_{p}^{sym}$ that is most right in Equation (43) constrains the basis $Y_{s}^{l,m}$ to m=0, whereas the operator $P_{p}^{sym}$ that is most left in Equation (43) constrains the basis $Y_{s}^{l^{\prime },m^{\prime }}$ to $m^{\prime }=0$.

**Lemma** **1.** *(axial symmetry) Let $\tilde{U}:G\to C$ be axially symmetric. Then,*

*1.*  *it relates to a unique function U:G/H→C via $U\left(x,n\right)=\tilde{U}\left(x,R_{n}\right)$;*
*2.*  *the matrix coefficients*
  $${\hat{U}}_{l,m,l^{\prime },m^{\prime }}^{p,s}={\left[F_{G}\tilde{U}\left(\sigma^{p,s}\right)\right]}_{l,m,l^{\prime },m^{\prime }}\text{of linear operator }F_{G}\tilde{U}\left(\sigma^{p,s}\right)$$
  *relative to the modified spherical harmonic basis $\left\{Y_{s}^{l,m}\right\}$ vanish if m≠0; and*
*3.*  *the matrix coefficients*
  $${\hat{U}}_{l,m,l^{\prime },m^{\prime }}^{p,s}={\left[F_{G/H}U\left({\bar{\sigma }}^{p,s}\right)\right]}_{l,m,l^{\prime },m^{\prime }}\text{of linear operator }F_{G/H}U\left({\bar{\sigma }}^{p,s}\right)$$
  *relative to the modified spherical harmonic basis $\left\{Y_{s}^{l,m}\right\}$ vanish if m≠0 or $m^{\prime }\neq 0$.*

*Conversely, if $\tilde{U}=F_{G}^{-1}\left(\hat{U}\right)$ and*

(44) $$\forall_{p>0}\forall_{l\in N_{0}}\forall_{s\in Z,\mathrm{with}|s|\leq l}\;\forall_{m^{\prime }\in Z,\mathrm{with}\left|m^{\prime }\right|\leq l}\;\forall_{m\neq 0}\;:\;{\hat{U}}_{l,m,l^{\prime },m^{\prime }}^{p,s}=0,$$

*then $\tilde{U}$ satisfies the axial symmetry in Equation (41).*

**Proof.**  Item 1: Uniqueness of *U* follows by the fact that the choice of $R_{n}$ of some rotation that maps a onto n does not matter. Indeed, $U\left(x,n\right)=\tilde{U}\left(x,R_{n}R_{a,\bar{\alpha }}\right)=\tilde{U}\left(x,R_{n}\right)$.

Item 2: Assumption Equation (41) can be rewritten as $\tilde{U}\left(g\right)=\tilde{U}\left(gh_{\bar{\alpha }}\right)$ for all $h_{\bar{\alpha }}\in H$, g∈G. This gives:

(45) $$\begin{array}{cc} {\hat{U}}_{l,m,l^{\prime },m^{\prime }}^{p,s} & ={\langle \;\left(F_{G}\tilde{U}\right)\left(Y_{s}^{l^{\prime },m^{\prime }}\left(p^{-1}\cdot \right)\right)\;,\;Y_{s}^{l,m}\left(p^{-1}\cdot \right)\;\rangle }_{L_{2}\left(pS^{2}\right)}\\ & =\int_{G}\tilde{U}\left(g\right)\;{\langle \;\sigma_{g^{-1}}^{p,s}Y_{s}^{l^{\prime },m^{\prime }}\left(p^{-1}\cdot \right)\;,\;Y_{s}^{l,m}\left(p^{-1}\cdot \right)\;\rangle }_{L_{2}\left(pS^{2}\right)}\;dg\\ & =\int_{G}\tilde{U}\left(g\right)\;{\langle \;Y_{s}^{l^{\prime },m^{\prime }}\left(p^{-1}\cdot \right)\;\;,\;\sigma_{g}^{p,s}Y_{s}^{l,m}\left(p^{-1}\cdot \right)\rangle }_{L_{2}\left(pS^{2}\right)}\;\;dg\\ & =\int_{G}\tilde{U}\left(gh_{\bar{\alpha }}\right)\;{\langle \;Y_{s}^{l^{\prime },m^{\prime }}\left(p^{-1}\cdot \right)\;,\;\sigma_{gh_{\bar{\alpha }}}^{p,s}Y_{s}^{l,m}\left(p^{-1}\cdot \right)\;\rangle }_{L_{2}\left(pS^{2}\right)}\;d\left(gh_{\bar{\alpha }}\right)\\ & =\int_{G}\tilde{U}\left(g\right)\;{\langle \;Y_{s}^{l^{\prime },m^{\prime }}\left(p^{-1}\cdot \right)\;,\;\sigma_{g}^{p,s}\circ \sigma_{h_{\bar{\alpha }}}^{p,s}Y_{s}^{l,m}\left(p^{-1}\cdot \right)\;\rangle }_{L_{2}\left(pS^{2}\right)}\;d\left(gh_{\bar{\alpha }}\right)\\ & =e^{-im\bar{\alpha }}\;{\hat{U}}_{l,m,l^{\prime },m^{\prime }}^{p,s}\mathrm{for}\mathrm{all}\bar{\alpha }\in \left[0,2\pi \right), \end{array}$$

where we recall that σ is a UIR and that the Haar measure on *G* is bi-invariant. In the first step, we used the third property, whereas in the final step we used the second property of Proposition 2 together with

(46) $$D_{m^{\prime }m}^{l}\left(R_{a,\bar{\alpha }}\right)=e^{-im\bar{\alpha }}\delta_{m^{\prime }m}\;\mathrm{so}\mathrm{that}\sigma_{h_{\bar{\alpha }}}^{p,s}Y_{s}^{l,m}\left(p^{-1}\cdot \right)=e^{-im\bar{\alpha }}Y_{s}^{l,m}\left(p^{-1}\cdot \right).$$

We conclude that $\left(1-e^{-im\bar{\alpha }}\right){\hat{U}}_{l,m,l^{\prime },m^{\prime }}^{p,s}=0$ for all $\bar{\alpha }\in \left[0,2\pi \right)$ so $m\neq 0\Rightarrow {\hat{U}}_{l,m,l^{\prime },m^{\prime }}^{p,s}=0$.

Item 3: Due to the second property in Proposition 2, we have

$$\sigma_{\left(0,R\right)}^{p,s}Y_{s}^{l,m}\left(p^{-1}\cdot \right)=\sum_{m^{\prime }=-l}^{l}D_{m^{\prime }m}^{l}\left(R\right)\;\;Y_{s}^{l,m^{\prime }}\left(p^{-1}\cdot \right).$$

Thereby, the projection $P_{p}^{sym}$ is given by

(47) $$P_{p}^{sym}\left(\sum_{l=0}^{\infty }\sum_{m=-l}^{l}\alpha_{l,m}Y_{s}^{l,m}\right)=\sum_{l=0}^{\infty }\alpha_{l,0}Y_{s}^{l,0}.$$

Now, the projection $P_{p}^{sym}$ that is applied first in Equation (43) filters out m=0 as the only possible nonzero component. The second projection filters out $m^{\prime }=0$ as the only possible nonzero component.

Conversely, if Equation (44) holds, one has by inversion Equation (35) that

$$\tilde{U}\left(g\right)=\frac{1}{2\pi^{2}}\sum_{s\in Z}\sum_{l=|s|}^{\infty }\sum_{l^{\prime }=\left|s\right|}^{\infty }\sum_{m^{\prime }=-l^{\prime }}^{l^{\prime }}\;\int_{0}^{\infty }{\hat{U}}_{l,0,l^{\prime },m^{\prime }}^{p,s}\;{\left(\sigma_{g}^{p,s}\right)}_{l^{\prime },m^{\prime },l,0}\;p^{2}dp,$$

so then the final result follows by the identity

(48) $${\left(\sigma_{gh_{\bar{\alpha }}}^{p,s}\right)}_{l^{\prime },m^{\prime },l,0}={\left(\sigma_{g}^{p,s}\right)}_{l^{\prime },m^{\prime },l,0}.$$

Thus, it remains to show why Equation (48) holds. It is due to $\sigma_{\left(x,R\right)}^{p,s}=\sigma_{\left(x,I\right)}^{p,s}\circ \sigma_{\left(0,R\right)}^{p,s}$ and Equation (46), as one has

(49) $$\begin{array}{c} \sigma_{gh_{\bar{\alpha }}}^{p,s}=\sigma_{\left(x,R\right)\left(0,R_{a,\bar{\alpha }}\right)}^{p,s}=\sigma_{\left(x,RR_{a,\bar{\alpha }}\right)}^{p,s}=\sigma_{\left(x,R\right)}^{p,s}\circ \sigma_{\left(0,R_{a,\bar{\alpha }}\right)}^{p,s},\mathrm{and}Y_{s}^{l,0}\left(p^{-1}R_{a,\bar{\alpha }}^{-1}\cdot \right)=Y_{s}^{l,0}\left(p^{-1}\cdot \right) \end{array}$$

and thereby Equation (48) follows by Equation (36). □

**Lemma** **2.** *If $\tilde{K}\in L_{2}\left(G\right)$ is real-valued and satisfies the axial symmetry in Equation (41), and moreover the following holds*

(50) $$\tilde{K}\left(g^{-1}\right)=\tilde{K}\left(g\right)$$

*then the Fourier coefficients satisfy ${\hat{K}}_{l,m,l^{\prime },m^{\prime }}^{p,s}=\bar{{\hat{K}}_{l^{\prime },m^{\prime },l,m}^{p,s}}$ and they vanish for m≠0 and for $m^{\prime }\neq 0$.*

**Proof.**  The proof follows by Equation (37) and inversion invariance of the Haar measure on *G* (see [86]). □

The next lemma shows that Equation (50) is a sufficient but not a necessary condition for the Fourier coefficients to vanish for both the cases $m^{\prime }\neq 0$ and m≠0.

**Lemma** **3.** *Let $\tilde{K}\in L_{2}\left(G\right)$ and $K\in L_{2}\left(G/H\right)$ be related by Equation (42). Then, we have the following equivalences:*

(51) $$\begin{array}{c} K\left(x,n\right)=K\left(R_{a,\bar{\alpha }}x,R_{a,\bar{\alpha }}n\right),\;\;\;\text{for all }\bar{\alpha }\in \left[0,2\pi \right),\left(x,n\right)\in G/H\\ ⇕\\ \tilde{K}\left(gh\right)=\tilde{K}\left(g\right)=\tilde{K}\left(hg\right),\;\;\;\text{for all }g\in G,\;h\in H\\ ⇕\\ \text{The Fourier coefficients }{\hat{K}}_{l,m,l^{\prime },m^{\prime }}^{p,s}\text{ vanish for }m\neq 0\text{ and for }m^{\prime }\neq 0. \end{array}$$

**Proof.**  We show a⇒b⇒c⇒a to get a⇔b⇔c.

a⇒b: Denoting $h=h_{\bar{\alpha }}=\left(0,R_{a,\bar{\alpha }}\right)$, g=(x,R), we have

$$\begin{array}{cc} \forall_{\bar{\alpha },{\bar{\alpha }}^{\prime }\in \left[0,2\pi \right)}\forall_{x\in R^{3}}\forall_{R\in SO(3)}\;:\;\tilde{K}\left(gh_{\bar{\alpha }}\right)\;\;\; & =\tilde{K}\left(x,RR_{a,\bar{\alpha }}\right)=K\left(x,RR_{a,\bar{\alpha }}a\right)=K\left(x,Ra\right)=\tilde{K}\left(x,R\right)=\tilde{K}\left(g\right)\\ & =K\left(R_{a,\bar{\alpha }}x,R_{a,\bar{\alpha }}Ra\right)=\tilde{K}\left(R_{a,\bar{\alpha }}x,R_{a,\bar{\alpha }}R\right)=\tilde{K}\left(h_{\bar{\alpha }}g\right). \end{array}$$

b⇒c: By Lemma 1, we know that the Fourier coefficients vanish for m≠0. Next, we show they also vanish for $m^{\prime }\neq 0$. Similar to Equation (49) we have

(52) $$\begin{array}{c} \sigma_{h_{\bar{\alpha }}g}^{p,s}=\sigma_{\left(R_{a,\bar{\alpha }}x,R_{a,\bar{\alpha }}R\right)}^{p,s}=\sigma_{\left(R_{a,\bar{\alpha }}x,I\right)}^{p,s}\circ \sigma_{\left(0,R_{a,\bar{\alpha }}R\right)}^{p,s}\;, \end{array}$$

which gives the following relation for the matrix-coefficients:

(53) $$\begin{array}{c} {\left(\sigma_{g=(x,R)}^{p,s}\right)}_{l^{\prime },m^{\prime },l,m}=\sum_{j=-l}^{l}{\langle \sigma_{\left(x,I\right)}^{p,s}Y_{s}^{l,j}\left(p^{-1}\cdot \right)\;,\;Y_{s}^{l^{\prime },m^{\prime }}\left(p^{-1}\cdot \right)\rangle }_{L_{2}\left(pS^{2}\right)}\;D_{jm}^{l}\left(R\right)\;\Rightarrow \\ {\left(\sigma_{h_{\bar{\alpha }}\;g}^{p,s}\right)}_{l^{\prime },m^{\prime },l,m}=\sum_{j=-l}^{l}e^{-i\left(m^{\prime }-j\right)\bar{\alpha }}{\langle \sigma_{\left(x,I\right)}^{p,s}Y_{s}^{l,j}\left(p^{-1}\cdot \right)\;,\;Y_{s}^{l^{\prime },m^{\prime }}\left(p^{-1}\cdot \right)\rangle }_{L_{2}\left(pS^{2}\right)}\;e^{-i\;j\bar{\alpha }}\;D_{jm}^{l}\left(R\right)\Rightarrow \\ {\left(\sigma_{h_{\bar{\alpha }}g}^{p,s}\right)}_{l^{\prime },m^{\prime },l,m}=e^{-im^{\prime }\bar{\alpha }}\;{\left(\sigma_{g}^{p,s}\right)}_{l^{\prime },m^{\prime },l,m}. \end{array}$$

The implication can be directly verified by Proposition 2, Equations (34) and (52), and

$$\begin{array}{cc} {\langle Y_{s}^{l^{\prime },m^{\prime }}\left(p^{-1}\cdot \right)\;,\;\sigma_{\left(R_{a,\bar{\alpha }}x,I\right)}^{p,s}Y_{s}^{l,j}\left(p^{-1}\cdot \right)\rangle }_{L_{2}\left(pS^{2}\right)} & =\int_{pS^{2}}e^{-ip(x\cdot R_{a,\bar{\alpha }}^{T}u)}\;Y_{s}^{l,j}\left(u\right)\;\bar{Y_{s}^{l^{\prime },m^{\prime }}\left(u\right)}\;d_{\mu_{pS^{2}}}\left(u\right)\\ & =\int_{pS^{2}}e^{-ip(x\cdot v)}Y_{s}^{l,j}\left(R_{a,\bar{\alpha }}v\right)\;\bar{Y_{s}^{l^{\prime },m^{\prime }}\left(R_{a,\bar{\alpha }}v\right)}\;d_{\mu_{pS^{2}}}\left(v\right). \end{array}$$

From Equation (53), we deduce that:

$$\begin{array}{cc} {\hat{K}}_{l,m,l^{\prime },m^{\prime }}^{p,s} & =\int_{G}\tilde{K}\left(g\right)\;{\langle \;\sigma_{g}^{p,s}Y_{s}^{l,m}\left(p^{-1}\cdot \right)\;,\;Y_{s}^{l^{\prime },m^{\prime }}\left(p^{-1}\cdot \right)\;\rangle }_{L_{2}\left(pS^{2}\right)}\;\;dg\\ & =\int_{G}\tilde{K}\left(h_{\bar{\alpha }}g\right)\;{\langle \;\sigma_{h_{\bar{\alpha }}g}^{p,s}Y_{s}^{l,m}\left(p^{-1}\cdot \right)\;,\;Y_{s}^{l^{\prime },m^{\prime }}\left(p^{-1}\cdot \right)\;\rangle }_{L_{2}\left(pS^{2}\right)}\;d\left(h_{\bar{\alpha }}g\right)\\ & =\int_{G}\tilde{K}\left(g\right)\;{\langle \;\sigma_{g}^{p,s}Y_{s}^{l,m}\left(p^{-1}\cdot \right)\;,\;\sigma_{h_{\bar{\alpha }}^{-1}}^{p,s}Y_{s}^{l^{\prime },m^{\prime }}\left(p^{-1}\cdot \right)\;\rangle }_{L_{2}\left(pS^{2}\right)}\;dg=e^{+im^{\prime }\;\bar{\alpha }}\;{\hat{K}}_{l,m,l^{\prime },m^{\prime }}^{p,s}, \end{array}$$

which holds for all $\bar{\alpha }\in \left[0,2\pi \right)$. Thereby, if $m^{\prime }\neq 0$, then ${\hat{K}}_{l,m,l^{\prime },m^{\prime }}^{p,s}=0$.

c⇒a: By inversion of Equation (35), where the only contributing terms have m=0 and $m^{\prime }=0$, we see that $\tilde{K}\left(gh\right)=\tilde{K}\left(hg\right)=\tilde{K}\left(g\right)$ for all $h=(0,R_{a,\bar{\alpha }})$. Thereby, $\tilde{K}$ is axially symmetric and by Lemma 1 it relates to a unique kernel on G/H via $K\left(x,n\right)=\tilde{K}\left(x,R_{n}\right)$ and the result follows by Equation (30). □

Now that we have characterized all functions $K\in L_{2}\left(G/H\right)$ for which the Fourier coefficients ${\hat{K}}_{l,m,l^{\prime },m^{\prime }}^{p,s}$ vanish for m≠0 and $m^{\prime }\neq 0$ in the above lemma, we considerably simplify the inversion and Plancherel formula for Fourier transform $F_{G}$ on the group G=SE(3) to the Fourier transform $F_{G/H}$ on the homogeneous space $G/H=R^{3}⋊S^{2}$ in the next theorem. This is important to our objective of deriving the kernels for the linear PDEs in Equation (6) that we address in the next section.

**Theorem** **1.** *(matrix-representation for $F_{G/H}$, explicit inversion and Plancherel formula)*

*Let $K\in L_{2}^{sym}\left(G/H\right)$ and $\tilde{K}\in L_{2}\left(G\right)$ be related by Equation (42). Then, the matrix elements of $F_{G/H}K$ are given by*

$$\begin{array}{cc} {\hat{K}}_{l^{\prime },0,l,0}^{p,s} & =\int_{G}\tilde{K}\left(g\right)\;{\left(\sigma_{g^{-1}}^{p,s}\right)}_{l^{\prime },0,l,0}\;dg\;,\\ \mathrm{with}{\left(\sigma_{g}^{p,s}\right)}_{l^{\prime },0,l,0} & =\sum_{j=-l}^{l}\;\left[l^{\prime },0\;\left|\;p,s\;\right|l,j\right]\left(x\right)\;D_{j0}^{l}\left(R\right)\;\;\;\text{ for all }g=\left(x,R\right)\in G. \end{array}$$

*The constants $\left[l^{\prime },0\;\left|\;p,s\;\right|l,j\right]\left(x\right):={\langle \;\sigma_{\left(x,I\right)}^{p,s}Y_{s}^{l,j}\left(p^{-1}\cdot \right)\;,\;Y_{s}^{l^{\prime },0}\left(p^{-1}\cdot \right)\;\rangle }_{L_{2}\left(pS^{2}\right)}$ admit an analytic expression in terms of elementary functions ([4], Equation10.34) and the Wigner D-functions in Equation (34).*

*Furthermore, we have the following Plancherel and inversion formula:*

$$\begin{array}{cc} {∥K∥}_{L_{2}\left(G/H\right)}^{2} & =∥F_{G/H}{K∥}^{2}=\sum_{s\in Z}\;\int_{R^{+}}∥\;|\left(F_{G/H}K\right)\left({\bar{\sigma }}^{p,s}\right){∥\;|}^{2}\;p^{2}dp=\int_{R^{+}}\sum_{s=-\infty }^{\infty }\sum_{l^{\prime }=\left|s\right|}^{\infty }\sum_{l=|s|}^{\infty }{\left|{\hat{K}}_{l,0,l^{\prime },0}^{p,s}\right|}^{2}\;p^{2}dp,\\ K(x,n) & =\left(F_{G/H}^{-1}F_{G/H}K\right)\left(x,n\right)=\sum_{s\in Z}\;\int_{R^{+}}\mathrm{trace}\left\{\left(F_{G/H}K\right)\left({\bar{\sigma }}^{p,s}\right)\;{\bar{\sigma }}_{\left(x,n\right)}^{p,s}\right\}\;p^{2}dp\\ & =\frac{1}{2\pi^{2}}\sum_{s\in Z}\sum_{l^{\prime }=\left|s\right|}^{\infty }\sum_{l=|s|}^{\infty }\;\int_{R^{+}}{\hat{K}}_{l,0,l^{\prime },0}^{p,s}\;{\left({\bar{\sigma }}_{\left(x,n\right)}^{p,s}\right)}_{l^{\prime },0,l,0}\;p^{2}dp, \end{array}$$

*with matrix coefficients given by (for analytic formulas, see ([4], eq.10.35))*

(54) $$\begin{array}{cc} {\left({\bar{\sigma }}_{\left(x,n\right)}^{p,s}\right)}_{l^{\prime },0,l,0} & ={\left(\sigma_{g}^{p,s}\right)}_{l^{\prime },0,l,0}={\langle \;\sigma_{g}^{p,s}Y_{s}^{l,0}\left(p^{-1}\cdot \right)\;,\;Y_{s}^{l^{\prime },0}\left(p^{-1}\cdot \right)\;\rangle }_{L_{2}\left(pS^{2}\right)}\\ & ={\langle \;\sigma_{g}^{p,s}Y^{l,s}\left(p^{-1}\cdot \right)\;,\;Y^{l^{\prime },s}\left(p^{-1}\cdot \right)\;\rangle }_{L_{2}\left(pS^{2}\right)}\;\;\text{for }g=\left(x,R_{n}\right). \end{array}$$

**Proof.**  The above formulas are a direct consequence of Lemma 3 and the Plancherel and inversion formulas (see [4], ch:10.8, [26]) for Fourier transform on SE(3). Recall that a coordinate-free definition of ${\bar{\sigma }}^{p,s}$ is given in Equation (40). Its matrix coefficients are given by Equation (54), where we recall the first item of Proposition 2 and where we note that they are independent on the choice of $R_{n}\in SO\left(3\right)$ mapping a onto n. □

**Corollary** **1.** *Let $K_{1}$, $K_{2}\in L_{2}^{sym}\left(G/H\right)$. Then, for shift-twist convolution on $G/H=R^{3}⋊S^{2}$ given by*

$$\left(K_{1}*K_{2}\right)\left(x,n\right)=\int_{S^{2}}\int_{R^{3}}K_{1}\left(R_{n^{\prime }}^{T}\left(x-x^{\prime }\right),R_{n^{\prime }}^{T}n\right)\;K_{2}\left(x^{\prime },n^{\prime }\right)\;dx^{\prime }d\mu_{S^{2}}\left(n^{\prime }\right)$$

*we have $F_{G/H}\left(K_{1}*K_{2}\right)=\left(F_{G/H}K_{1}\right)\circ \left(F_{G/H}K_{2}\right).$*

**Proof.**  Set ${\tilde{K}}_{1}\left(g\right)\;=\;K_{1}\left(g⊙\left(0,a\right)\right)$. Standard Fourier theory [5] gives $F_{G}\left(\tilde{K_{1}*K_{2}}\right)\;=\;F_{G}\left({\tilde{K}}_{1}*{\tilde{K}}_{2}\right)$, so

$$\begin{array}{ccc} F_{G/H}\left(K_{1}*K_{2}\right) & \overset{\mathrm{def}}{=} & P_{p}^{sym}\circ F_{G}\left(\tilde{K_{1}*K_{2}}\right)\circ P_{p}^{sym}\\ & = & P_{p}^{sym}\circ F_{G}\left({\tilde{K}}_{1}\right)\circ F_{G}\left({\tilde{K}}_{2}\right)\circ P_{p}^{sym}\\ & = & P_{p}^{sym}\circ F_{G}\left({\tilde{K}}_{1}\right)\circ P_{p}^{sym}\circ P_{p}^{sym}\circ F_{G}\left({\tilde{K}}_{2}\right)\circ P_{p}^{sym}\\ & = & \left(F_{G/H}K_{1}\right)\circ \left(F_{G/H}K_{2}\right), \end{array}$$

where the first equality is given by Equation (43) and the third equality follows by Lemma 3 and Equation (47). □

## 5. Application of the Fourier Transform on $R^{3}⋊S^{2}$ for Explicit Solutions of the Fokker–Planck PDEs of α-stable Lévy Processes on $R^{3}⋊S^{2}$

Our objective is to solve the PDE system in Equation (6) on the homogeneous space of positions and orientations G/H. Recall that we extended this PDE system to *G* in Equation (10). As the cases $D_{11}>0$ follow from the case $D_{11}=0$ (recall Section 2.2), we consider the case $D_{11}=0$ in this section. From the symmetry consideration in Section 2, it follows that the solution of Equation (10) is given by ${\tilde{W}}_{\alpha }\left(g,t\right)=\left({\tilde{K}}_{t}^{\alpha }*\tilde{U}\right)\left(g\right)$ with a probability kernel ${\tilde{K}}_{t}^{\alpha }:G\to R^{+}$, whereas the solution of Equation (6) is given by

$$W_{\alpha }\left(x,n,t\right)=\left(K_{t}^{\alpha }*U\right)\left(x,n\right):=\int_{S^{2}}\int_{R^{3}}K_{t}^{\alpha }\left(R_{n^{\prime }}^{T}\left(x-x^{\prime }\right),R_{n^{\prime }}^{T}n\right)\;U\left(x^{\prime },n^{\prime }\right)\;dx^{\prime }d\mu_{S^{2}}\left(n^{\prime }\right),$$

where the kernels $K_{t}^{\alpha }$ are invariant with respect to left-actions of the subgroup *H* (recall Equation (30)). This invariance means that the condition for application of the Fourier transform $F_{G/H}$ on $R^{3}⋊S^{2}$ is satisfied (recall Lemma 3) and we can indeed employ Theorem 1 to keep all our computations, spectral decompositions and Fourier transforms in the 5D homogeneous space $R^{3}⋊S^{2}=G/H$ rather than a technical and less direct approach [40] in the 6D group G=SE(3).

**Remark** **8.** *For the underlying probability theory, and sample paths of discrete random walks of the α-Stable Lévy stochastic processes, we refer to Appendix A. To get a general impression of how Monte Carlo simulations of such stochastic processes can be used to approximate the exact probability kernels $K_{t}^{\alpha }$, see Figure 1. In essence, such a stochastic approximation is computed by binning the endpoints of the random walks. A brief mathematical explanation follows in Section 5.2.*

For now, let us ignore the probability theory details and let us first focus on deriving exact analytic solutions to Equation (6) and its kernel $K_{t}^{\alpha }$ via Fourier transform $F_{G/H}$ on $G/H=R^{3}⋊S^{2}$.

### 5.1. Exact Kernel Representations by Spectral Decomposition in the Fourier Domain

Let us consider the evolution in Equation (6) for α-stable Lévy process on the quotient $G/H=R^{3}⋊S^{2}$. Then, the mapping from the initial condition $W\left(\cdot ,0\right)=U\left(\cdot \right)\in L_{2}\left(G/H\right)$ to the solution W(·,t) at a fixed time t≥0 is a bounded linear mapping. It gives rise to a strongly continuous (holomorphic) semigroup [66]. We conveniently denote the bounded linear operator on $L_{2}\left(G/H\right)$ as follows:

(55) $$W_{\alpha }\left(\cdot ,t\right)=\left(e^{tQ_{\alpha }}U\right)\left(\cdot \right),\;\mathrm{for}\mathrm{all}t\geq 0.$$

In the next main theorem, we provide a spectral decomposition of the operator using both a direct sum and a direct integral decomposition. Note that definitions of direct integral decompositions (and the underlying measure theory) can be found in ([24], ch:3.3 and 3.4).

#### 5.1.1. Eigenfunctions and Preliminaries

To formulate the main theorem, we need some preliminaries and formalities. First, let us define ${\bar{F}}_{R^{3}}:L_{2}\left(R^{3}⋊S^{2}\right)\to L_{2}\left(R^{3}⋊S^{2}\right)$ by

(56) $$\left({\bar{F}}_{R^{3}}U\right)\left(\omega ,n\right):=\left[F_{R^{3}}U\left(\cdot ,n\right)\right]\left(\omega \right).$$

**Figure 2:**
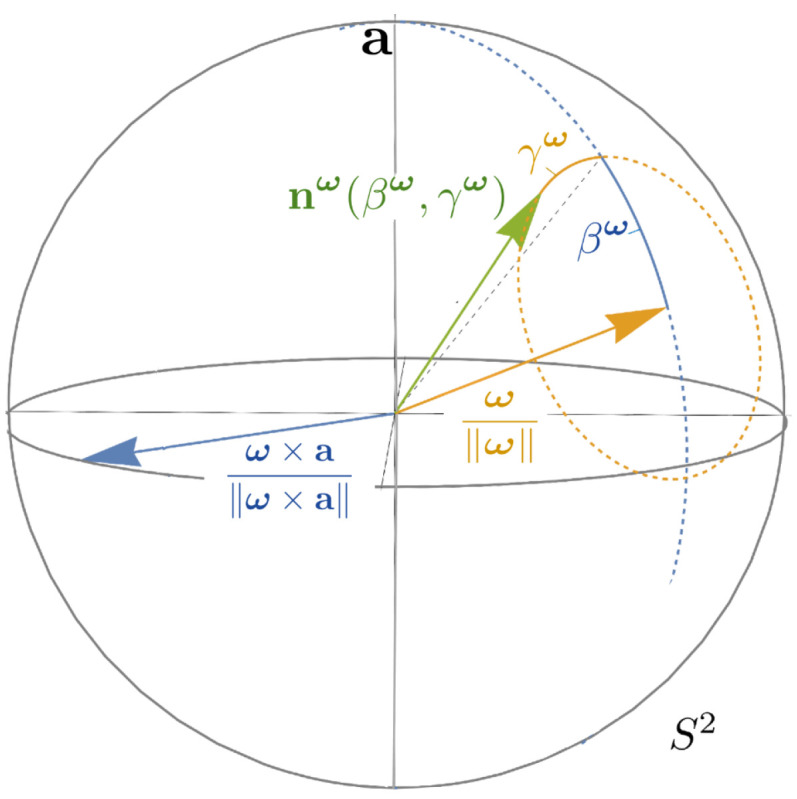
For ω≠a, we parameterize every orientation n (green) by rotations around $r^{-1}\omega$ (orange) and $\frac{\omega \times a}{\left||\omega \times a|\right|}$ (blue). In other words, $n^{\omega }\left(\beta^{\omega },\gamma^{\omega }\right)=R_{r^{-1}\omega ,\gamma^{\omega }}R_{\frac{\omega \times a}{\left||\omega \times a|\right|},\beta^{\omega }}\left(r^{-1}\omega \right)$.

Recalling Equation (19), we re-express the generator in the spatial Fourier domain:

(57) $$\begin{array}{cc} -{\left(-B\right)}^{\alpha } & ={\bar{F}}_{R^{3}}\circ Q_{\alpha }\circ {\bar{F}}_{R^{3}}^{-1}\Rightarrow \\ -{\left(-B_{\omega }\right)}^{\alpha } & =-{\left(-D_{33}\;{\left(i\omega \cdot n\right)}^{2}-D_{44}\;\Delta_{n}^{S^{2}}\right)}^{\alpha }\\ & =-{\left(D_{33}\;r^{2}\;{\left(a\cdot (R_{r^{-1}\omega }^{T}n)\right)}^{2}-D_{44}\;\Delta_{n}^{S^{2}}\right)}^{\alpha }\\ & =-{\left(D_{33}\;r^{2}\;\cos^{2}\left(\beta^{\omega }\right)-D_{44}\;\Delta_{n}^{S^{2}}\right)}^{\alpha },\;\;\mathrm{with}r=∥\omega ∥,\alpha \in \left(0,1\right], \end{array}$$

where $\beta^{\omega }$ denotes the angle between n and $r^{-1}\omega$ (see Figure 2). This re-expression is the main reason for the following definitions.

Instead of the modified spherical Harmonics $Y_{s}^{l,m}$ in Proposition 2, which are commonly used as a standard basis to represent each operator in the Fourier transform on SE(3), we use our generalized spherical harmonics, depending on a spatial frequency vector, as this is in accordance with Equation (57).

**Definition** **10.** *Let $l\in N_{0}$. Let m∈Z such that |m|≤l. Let $\omega \in R^{3}$ be a frequency vector. We define*

(58) $$Y_{\omega }^{l,m}\left(n\right)=Y^{l,m}\left(R_{r^{-1}\omega }^{T}n\right),\;\mathrm{with}r=∥\omega ∥,\;\;n\in S^{2},$$

*where we take the rotation which maps a onto $r^{-1}\omega$ whose matrix representation in the standard basis is:*

$$R_{r^{-1}\omega }=\left(\begin{array}{ccccc} \frac{(\omega \times a)\times \omega }{\left||(\omega \times a)\times \omega |\right|} & | & \frac{\omega \times a}{\left||\omega \times a|\right|} & | & r^{-1}\omega \; \end{array}\right)\mathrm{for}r^{-1}\omega \neq a,\text{and }R_{a}=I,\text{and }R_{0}=I.$$

Recall the standard spherical angle formula $n\left(\beta ,\gamma \right)={\left(\sin\beta \cos\gamma ,\sin\beta \sin\gamma ,\cos\beta \right)}^{T}$ from Proposition 2. These are Euler-angles relative to the reference axis $a=e_{z}$. For the Euler-angles relative to the (normalized) frequency $r^{-1}\omega$ one has (see also Figure 2):

(59) $$n^{\omega }\left(\beta^{\omega },\gamma^{\omega }\right)=R_{r^{-1}\omega }n\left(\beta^{\omega },\gamma^{\omega }\right).$$

**Definition** **11.** *Let $l\in N_{0}$. Let m∈Z such that |m|≤l. We define the functions $\Phi_{\omega }^{l,m}\in L_{2}\left(S^{2}\right)$ by*

(60) $$\Phi_{\omega }^{l,m}\left(n\right)=\sum_{j=0}^{\infty }\frac{d_{j}^{l,m}\left(r\right)}{∥d^{l,m}\left(r\right)∥}\;Y_{\omega }^{|m|+j,m}\left(n\right),$$

*where r=∥ω∥ and $d^{l,m}\left(r\right):={\left(d_{j}^{l,m}\left(r\right)\right)}_{j=0}^{\infty }$ are coefficients such that*

$$\Phi_{\omega }^{l,m}\left(n^{\omega }\left(\beta^{\omega },\gamma^{\omega }\right)\right)=S_{\rho }^{l,m}\left(\cos\beta^{\omega }\right)\;\frac{e^{im\gamma^{\omega }}}{\sqrt{2\pi }},\mathrm{with}\rho =r\sqrt{\frac{D_{33}}{D_{44}}},$$

*where $S_{\rho }^{l,m}\left(\cdot \right)$ denotes the $L_{2}$-normalized spheroidal wave function.*

**Remark** **9.** *The spheroidal wave function arises from application of the method of separation on operator $B_{\omega }$ in Equation (57) where basic computations (for details, see [40]) lead to the following singular Sturm-Liouville problem:*

(61) $$\left(Ly\right)\left(x\right)=\frac{d}{dx}\left[p\left(x\right)\frac{dy(x)}{dx}\right]+q\left(x\right)y\left(x\right)=-\lambda \left(r\right)\;y\left(x\right),\;x=\cos\beta^{\omega }\in \left[-1,1\right].$$

*with $p\left(x\right)=\left(1-x^{2}\right)$, $q\left(x\right)=-\rho^{2}x^{2}-\frac{m^{2}}{1-x^{2}}$, and again $\rho =r\sqrt{D_{33}/D_{44}}$. In this formulation, p(x) vanishes at the boundary of the interval, which makes our problem a singular Sturm–Liouville problem. It is sufficient to require boundedness of the solution and its derivative at the boundary points to have nonnegative, distinct, simple eigenvalues $\lambda_{r}^{l,m}$ and existence of a countable, complete orthonormal basis of eigenfunctions ${\left\{y_{j}\right\}}_{j=0}^{\infty }$ [91] for the spheroidal wave equation.*

*As a result, standard Sturm–Liouville theory (that applies the spectral decomposition theorem for compact self-adjoint operators to a kernel operator that is the right-inverse of L) provides us (for each ω fixed) a complete orthonormal basis of eigenfunctions $\left\{\Phi_{\omega }^{l,m}\right\}$ in $L_{2}\left(S^{2}\right)$ with eigenvalues of our (unbounded) generators:*

(62) $$-{\left(-B_{\omega }\right)}^{\alpha }\Phi_{\omega }^{l,m}=-{\left(-\lambda_{r}^{l,m}\right)}^{\alpha }\;\Phi_{\omega }^{l,m},\;\text{for all }\left|m\right|\leq l.$$

**Remark** **10.** *Define $Y_{l,m}\left(\beta ,\gamma \right):=Y^{l,m}\left(n\left(\beta ,\gamma \right)\right)$. Then, Equations (58) and (59) imply $Y_{\omega }^{l,m}\left(n^{\omega }\left(\beta^{\omega },\gamma^{\omega }\right)\right)=Y_{l,m}\left(\beta^{\omega },\gamma^{\omega }\right)$.*

**Remark** **11.** *The matrix-representation of $-{\left(-B_{\omega }\right)}^{\alpha }$ with respect to orthonormal basis ${\left\{Y_{\omega }^{|m|+j,m}\right\}}_{j\in N_{0},m\in Z}$ equals*

$$\underset{m\in Z}{⨁}-{\left(D_{33}r^{2}M^{m}+D_{44}\Lambda^{m}\right)}^{\alpha },$$

*where $\Lambda^{m}:=\mathrm{diag}{\left\{l\left(l+1\right)\right\}}_{l=|m|}^{\infty }=\mathrm{diag}\{(|m|+j)(|m|+j+{1)\}}_{j=0}^{\infty }$, r=∥ω∥ and where $M^{m}$ is the tri-diagonal matrix (that can be computed analytically ([40], eq. 106)) given by*

(63) $${\left(\cos\beta \right)}^{2}Y^{|m|+j,m}\left(n\left(\beta ,\gamma \right)\right)=\sum_{j^{\prime }=0}^{\infty }{\left({\left(M^{m}\right)}^{T}\right)}_{j,j^{\prime }}Y^{\left|m\right|+j^{\prime },m}\left(n\left(\beta ,\gamma \right)\right).$$

*As a result, we see from Equations (60) and (62) that the coefficients $d^{l,m}\left(r\right)$ for our eigenfunctions are eigenvectors of a matrix*

(64) $$-\left(D_{33}r^{2}M^{m}+D_{44}\Lambda^{m}\right)d^{l,m}\left(r\right)=\lambda_{r}^{l,m}d^{l,m}\left(r\right),\;\text{for }l\geq \left|m\right|.$$

*This matrix (and its diagonalization) play a central role for our main spectral decomposition theorem both in the spatial Fourier domain and in the Fourier domain of the homogeneous space of positions and orientations.*

#### 5.1.2. The Explicit Spectral Decomposition of the Evolution Operators

In Theorem 2, we present the explicit spectral decompositions both in the spatial Fourier domain and in the Fourier domain of the homogeneous space of positions and orientations.

Prior to this theorem, we explain the challenges that appear when we apply $F_{G/H}$ to the PDE of interest in Equation (6) on the quotient G/H. To get a grip on the evolution operator and the corresponding kernel, we set the initial condition equal to a delta distribution at the origin, i.e., we consider

$$U=\delta_{\left(0,a\right)}\Rightarrow W_{\alpha }\left(\cdot ,t\right)=e^{tQ_{\alpha }}U=e^{-t{\left(-Q\right)}^{\alpha }}\delta_{\left(0,a\right)}=K_{t}^{\alpha }.$$

In this case, the necessary condition in Equation (51) in Lemma 3 for application of $F_{G/H}$ is indeed satisfied, due to the symmetry property of the kernel, given by Equation (30). Now, due to linearity

$$F_{G/H}\circ e^{tQ_{\alpha }}\circ F_{G/H}^{-1}=e^{t\;\left(F_{G/H}\;\circ \;Q_{\alpha }\;\circ \;F_{G/H}^{-1}\right)},$$

we just need to study the generator in the Fourier domain.

For the moment, we set α=1 (the degenerate diffusion case) and return to the general case later on (recall Section 1.6 and Section 2.2). Then, it follows that (for details, see ([40], App.D))

(65) $$\begin{array}{c} \left(F_{G/H}\circ Q\circ F_{G/H}^{-1}{\hat{K}}_{t}^{1}\right)\left({\bar{\sigma }}^{p,s}\right)=\left(-D_{33}\;{\left(a\cdot u\right)}^{2}+D_{44}\;\Delta_{u}^{pS^{2}}\right){\hat{K}}_{t}^{1}\left({\bar{\sigma }}^{p,s}\right),\\ \mathrm{with}\mathrm{the}\mathrm{kernel}{\hat{K}}_{t}^{1}:=F_{G/H}K_{t}^{1}\left(\cdot \right). \end{array}$$

Here, $\Delta_{u}^{pS^{2}}$ denotes the Laplace–Beltrami operator on a sphere $pS^{2}\;=\;\{u\in R^{3}\left|∥u∥=p\right\}$ of radius p>0.

We recall that $u\in pS^{2}$ is the variable of the functions on which ${\bar{\sigma }}^{p,s}$ acts. Recalling Equation (32), the first part in the righthand side of Equation (65) denotes a multiplier operator M given by

$$\left(M\phi \right)\left(u\right):=-\;{\left(a\cdot u\right)}^{2}\phi \left(u\right),\mathrm{for}\mathrm{all}\phi \in L_{2}\left(pS^{2}\right),\mathrm{and}\mathrm{almost}\mathrm{every}u\in pS^{2}.$$

As a result, we obtain the following PDE system for ${\hat{K}}_{t}^{\alpha }$ (now for general α∈(0,1]):

$$\begin{array}{c} \left\{\begin{array}{cc} \frac{\partial }{\partial t}{\hat{K}}_{t}^{\alpha }\left({\bar{\sigma }}^{p,s}\right) & =-{\left(-D_{33}\;M-D_{44}\;\Delta_{u}^{pS^{2}}\right)}^{\alpha }{\hat{K}}_{t}^{\alpha }\left({\bar{\sigma }}^{p,s}\right)\\ {\hat{K}}_{0}^{\alpha }\left({\bar{\sigma }}^{p,s}\right) & =1_{L_{2}\left(pS^{2}\right)}. \end{array}\right. \end{array}$$

**Remark** **12.** *There is a striking analogy between the operators $F_{G/H}\circ Q_{\alpha }\circ F_{G/H}^{-1}$ and ${\bar{F}}_{R^{3}}\circ Q_{\alpha }\circ {\bar{F}}_{R^{3}}^{-1}$ given by Equation (57), where the role of $rR_{\omega /r}^{T}n$ corresponds to u. This correspondence ensures that the multipliers of the multiplier operators in the generator coincide and that the roles of p and r coincide:*

$$u=rR_{r^{-1}\omega }^{T}n\Rightarrow {\left(a\cdot u\right)}^{2}=r^{2}{\left(R_{r^{-1}\omega }^{T}a\cdot n\right)}^{2}={\left(\omega \cdot n\right)}^{2}\;\;\mathrm{and}\;\;∥u∥=p=r=∥\omega ∥.$$

**Lemma** **4.** *Let t≥0 and p>0 be fixed. The matrix-representation of operator $e^{t(D_{33}\;M+D_{44}\;\Delta_{u}^{pS^{2}})}:L_{2}\left(pS^{2}\right)\to L_{2}\left(pS^{2}\right)$ with respect to the orthonormal basis of spherical harmonics ${\left\{Y^{l=|s|+j\;,\;s}\left(p^{-1}\cdot \right)\right\}}_{j\in N_{0}\;,\;s\in Z}$ equals*

(66) $$\underset{s\in Z}{⨁}e^{-t\;(D_{33}p^{2}M^{s}+D_{44}\Lambda^{s})}\;.$$

**Proof.**  Recall Equation (63) that defines matrix $M^{m}$ (for analytic formulas of this tri-diagonal matrix, see [40]). This may be re-written as follows:

$${\left(a\cdot n\right)}^{2}Y^{|m|+j,m}\left(n\right)=\sum_{j^{\prime }=0}^{\infty }{\left({\left(M^{m}\right)}^{T}\right)}_{j,j^{\prime }}Y^{\left|m\right|+j^{\prime },m}\left(n\right).$$

Now, fix s∈Z and set m=s and $n=p^{-1}u$ and we have:

$${\langle \left(D_{33}M+D_{44}\;\Delta_{u}^{pS^{2}}\right)Y^{l,s}\left(p^{-1}\cdot \right)\;,\;Y^{l^{\prime },s}\left(p^{-1}\cdot \right)\;\rangle }_{L_{2}\left(pS^{2}\right)}=-p^{2}\;D_{33}{\left(M^{s}\right)}_{j^{\prime },j}-D_{44}l\left(l+1\right)\delta_{jj^{\prime }},$$

where again l=|s|+j, $l^{\prime }=\left|s\right|+j^{\prime }$ and $j,j^{\prime }\in N_{0}$.

Finally, we note that operator $D_{33}\;M+D_{44}\;\Delta_{u}^{pS^{2}}$ is negative definite and maps each subspace $\mathrm{span}\left\{{\left\{Y^{l,s}\left(p^{-1}\cdot \right)\right\}}_{l=|s|}^{\infty }\right\}$ for fixed s∈Z onto itself, which explains direct sum decomposition in Equation (66). □

Next, we formulate the main result, where we apply a standard identification of tensors a⊗b with linear maps:

(67) $$x\mapsto \left(a⊗b\right)\left(x\right)=\langle x\;,\;b\rangle a.$$

**Theorem** **2.** *We have the following spectral decompositions for the Forward-Kolomogorov evolution operator of α-stable Lévy-processes onthe homogeneous space $G/H=R^{3}⋊S^{2}$:*

- *In the Fourier domain of the homogeneous space of positions and orientations, we have:*
  (69) $$\begin{array}{c} \begin{array}{c} F_{G/H}\circ e^{-t{\left(-Q\right)}^{\alpha }}\circ F_{G/H}^{-1}\\ \;=\int_{R^{+}}^{⊕}\underset{s\in Z}{⨁}\sum_{l,l^{\prime }=\left|s\right|}^{\infty }{\left[e^{-{\left(D_{33}p^{2}M^{s}+D_{44}\Lambda^{s}\right)}^{\alpha }t}\right]}_{l,l^{\prime }}\left(Y^{l,s}\left(p^{-1}\cdot \right)⊗Y^{l^{\prime },s}\left(p^{-1}\cdot \right)\right)\;p^{2}dp\\ \;=\int_{R^{+}}^{⊕}\underset{s\in Z}{⨁}\sum_{l=|s|}^{\infty }\;e^{-{\left(-\lambda_{p}^{l,s}\right)}^{\alpha }t}\;\left(\Phi_{pa}^{l,s}\left(p^{-1}\cdot \right)⊗\Phi_{pa}^{l,s}\left(p^{-1}\cdot \right)\right)\;p^{2}dp \end{array} \end{array}$$
- *In the spatial Fourier domain, we have*
  (69) $$\begin{array}{c} \begin{array}{c} \left({\bar{F}}_{R^{3}}\circ e^{-t{\left(-Q\right)}^{\alpha }}\circ {\bar{F}}_{R^{3}}^{-1}\bar{U}\right)\left(\omega ,\cdot \right)=\bar{W}\left(\omega ,\cdot ,t\right)\\ \;=\sum_{m\in Z}\sum_{l,l^{\prime }=\left|m\right|}^{\infty }{\left[e^{-{\left(D_{33}r^{2}M^{m}+D_{44}\Lambda^{m}\right)}^{\alpha }t}\right]}_{l,l^{\prime }}\left(Y_{\omega }^{l,m}⊗Y_{\omega }^{l^{\prime },m}\right)\left(\bar{U}\left(\omega ,\cdot \right)\right)\\ \;=\sum_{m\in Z}\sum_{l=|m|}^{\infty }e^{-{\left(-\lambda_{r}^{l,m}\right)}^{\alpha }t}\;\left(\Phi_{\omega }^{l,m}⊗\Phi_{\omega }^{l,m}\right)\left(\bar{U}\left(\omega ,\cdot \right)\right) \end{array} \end{array}$$
  *where $\bar{W}\left(\omega ,\cdot ,t\right)={\bar{F}}_{R^{3}}W\left(\omega ,\cdot ,t\right)$ and $\bar{U}\left(\omega ,\cdot \right)={\bar{F}}_{R^{3}}U\left(\omega ,\cdot \right)$ (recall Equation (56)).*

*In both cases, the normalized eigenfunctions $\Phi_{\omega }^{l,m}$ are given by Equation (60) in Definition 11. The eigenvalues $\lambda_{r}^{l,m}$ are the eigenvalues of the spheroidal wave equation, as explained in Remark 9.*

**Proof.**  The first identity in Equation (68) follows by:

$$\begin{array}{cc} F_{G/H}\circ e^{-t{\left(-Q\right)}^{\alpha }}\circ F_{G/H}^{-1}= & e^{t\left(F_{G/H}\circ -{\left(-Q\right)}^{\alpha }\circ F_{G/H}^{-1}\right)}\\ \overset{([40],\mathrm{App}.D)\text{ and Theorem }1\;}{=} & \int_{R^{+}}^{⊕}e^{-t{\left(-D_{33}M+D_{44}\Delta_{u}^{pS^{2}}\right)}^{\alpha }}p^{2}dp\\ \overset{\text{Lemma }4\;\;\text{ and Theorem }1\;}{=} & \int_{R^{+}}^{⊕}\underset{s\in Z}{⨁}\sum_{l,l^{\prime }=\left|s\right|}^{\infty }{\left[e^{-t{\left(D_{33}p^{2}M^{s}+D_{44}\Lambda^{s}\right)}^{\alpha }}\right]}_{l,l^{\prime }}\left(Y^{l,s}\left(p^{-1}\cdot \right)⊗Y^{l^{\prime },s}\left(p^{-1}\cdot \right)\right)\;p^{2}dp\\ \overset{\left(60\right)}{=} & \int_{R^{+}}^{⊕}\underset{s\in Z}{⨁}\sum_{l=|s|}^{\infty }e^{-{\left(-\lambda_{p}^{l,s}\right)}^{\alpha }t}\;\left(\Phi_{pa}^{l,s}\left(p^{-1}\cdot \right)⊗\Phi_{pa}^{l,s}\left(p^{-1}\cdot \right)\right)\;p^{2}dp\;. \end{array}$$

In the last equality, we use the fact that $\Phi_{a}^{l,m}=Y^{l,m}$. By applying the identification in Equation (67), one observes that Equation (69) is a reformulation of Equation (24), was already been derived for α=1 in previous work by the first author with J.M. Portegies ([40], Thm.2.3 and Equation31). The key idea behind the derivation, the expansion and the completeness of the eigenfunctions $\left\{\Phi_{\omega }^{l,m}\right\}$ is summarized in Remark 9. The general case α∈(0,1] then directly follows by Section 1.6. □

Recently, exact formulas for the (degenerate) heat-kernels on G=SE(3) and on $G/H=R^{3}⋊S^{2}$ (i.e., the case α=1) have been published in [40]. In the next theorem:

- We extend these results to the kernels of PDE in Equation (6), which are Forward-Kolmogorov equations of α-stable Lévy process with α∈(0,1].
- We provide a structured alternative formula via the transform $F_{G/H}$ characterized in Theorem 1.

**Theorem** **3.** *We have the following formulas for the probability kernels of α-stable Lévy processes on $R^{3}⋊S^{2}$:*

- *Via conjugation with $F_{R^{3}⋊S^{2}}$:*
  (70) $$\begin{array}{c} K_{t}^{\alpha }\left(x,n\right)=\frac{1}{{\left(2\pi \right)}^{2}}\int_{0}^{\infty }\sum_{s\in Z}\sum_{l=|s|}^{\infty }e^{-{\left(-\lambda_{p}^{l,s}\right)}^{\alpha }t}\;{\left[{\bar{\sigma }}_{\left(x,n\right)}^{p,s}\right]}_{l,0,l,0}\;p^{2}dp, \end{array}$$
  *where* ${\left[{\bar{\sigma }}_{\left(x,n\right)}^{p,s}\right]}_{l,0,l,0}={\langle \sigma_{\left(x,R_{n}\right)}^{p,s}\Phi_{pa}^{l,s}\left(p^{-1}\cdot \right)\;,\;\Phi_{pa}^{l,s}\left(p^{-1}\cdot \right)\rangle }_{L_{2}\left(pS^{2}\right)}$ *can be derived analytically (see ([86], Rem. 16)).*
- *Via conjugation with ${\bar{F}}_{R^{3}}$:*
  (71) $$\begin{array}{c} K_{t}^{\alpha }\left(x,n\right)=\frac{1}{{\left(2\pi \right)}^{3}}\int_{R^{3}}\left(\sum_{l=0}^{\infty }\sum_{m=-l}^{l}e^{-{\left(-\lambda_{∥\omega ∥}^{l,m}\right)}^{\alpha }t}\;\bar{\Phi_{\omega }^{l,m}\left(a\right)}\;\Phi_{\omega }^{l,m}\left(n\right)\right)\;e^{ix\cdot \omega }\;d\omega . \end{array}$$

**Proof.**  Equation (70) follows by

$$K_{t}^{\alpha }\left(x,n\right)=\left(e^{tQ_{\alpha }}\delta_{\left(0,a\right)}\right)\left(x,n\right)=\left(F_{G/H}^{-1}\circ e^{tF_{G/H}\circ Q_{\alpha }\circ F_{G/H}^{-1}}\circ F_{G/H}\delta_{\left(0,a\right)}\right)\left(x,n\right).$$

Now, $\left(F_{G/H}\delta_{\left(0,a\right)}\right)\left(\sigma^{p,s}\right)=1_{L_{2}\left(pS^{2}\right)}$ implies $\left(\left(F_{G/H}\delta \right){\left(\sigma^{p,s}\right)}_{\left(0,a\right)}\right)\left(\sigma^{p,s}\right){)}_{l,0,l^{\prime },0}=\delta_{ll^{\prime }}$ so that the result follows by setting $U=\delta_{\left(0,a\right)}$ (or, more precisely, by taking *U* a sequence that is a bounded approximation of the unity centered around (0,a)) in Theorem 2, where we recall the inversion formula from the first part of Theorem 1.

Equation (71) follows similarly by

$$K_{t}^{\alpha }\left(x,n\right)=\left(e^{tQ_{\alpha }}\delta_{\left(0,a\right)}\right)\left(x,n\right)=\left({\bar{F}}_{R^{3}}^{-1}\circ e^{t{\bar{F}}_{R^{3}}\circ Q_{\alpha }\circ {\bar{F}}_{R^{3}}^{-1}}\circ {\bar{F}}_{R^{3}}\delta_{\left(0,a\right)}\right)\left(x,n\right).$$

Now, $\left({\bar{F}}_{R^{3}}\delta_{\left(0,a\right)}\right)\left(\sigma^{p,s}\right)=\frac{1}{{\left(2\pi \right)}^{\frac{3}{2}}}\delta_{a}$ and the result follows from the second part of Theorem 1 (again by taking *U* a sequence that is a bounded approximation of the unity centered around (0,a)). □

**Remark** **13.** *There also exist Gaussian estimates for the heat kernel $K_{t}^{\alpha =1}$ that use a weighted modulus on the logarithm on G [92]. Such Gaussian estimates can account for the quotient structure G/H [87], and can be reasonably close (cf. [44], Figure 4.4, [93]) to the exact solutions for practical parameter settings in applications [48,94,95].*

### 5.2. Monte-Carlo Approximations of the Kernels

A stochastic approximation for the kernel $K_{t}^{\alpha }$ is computed by binning the endpoints of discrete random walks simulating α-stable processes on the quotient $R^{3}⋊S^{2}$ that we explain next. Let us first consider the case α=1. For M∈N fixed, we have the discretization

(72) $$\left\{\begin{array}{c} X_{M}=X_{0}+\sum_{k=1}^{M}\sqrt{\frac{tD_{33}}{M}}\epsilon_{k}N_{k-1},\\ N_{M}=\left(\prod_{k=1}^{M}R_{a,\gamma_{k}}R_{e_{y},\beta_{k}\sqrt{\frac{tD_{44}}{M}}}\right)N_{0}=\left(R_{a,\gamma_{M}}R_{e_{y},\beta_{M}\sqrt{\frac{tD_{44}}{M}}}\circ \ldots \circ R_{a,\gamma_{1}}R_{e_{y},\beta_{1}\sqrt{\frac{tD_{44}}{M}}}\right)\;N_{0}\;, \end{array}\right.$$

with $\epsilon_{k}∼G_{t=1}^{R}∼N\left(0,\sigma =\sqrt{2}\right)$ stochastically independent Gaussian distributed on R with t=1; with uniformly distributed $\gamma_{k}∼\mathrm{Unif}\left(R/(2\pi Z)\equiv [-\pi ,\pi )\right)$; and $\beta_{k}∼g$, where $g:R\to R^{+}$ equals $g\left(r\right)=\frac{\left|r\right|}{2}\;e^{-\frac{r^{2}}{4}}$ in view of the theory of isotropic stochastic processes on Riemannian manifolds by Pinsky [96]. By the central limit theorem for independently distributed variables *with finite variance* it is only the variances of the distributions for the random variables *g* and $G_{t=1}^{R}$ that matter. One may also take

$$\begin{array}{c} \epsilon_{k}∼\sqrt{3}\;\mathrm{Unif}\left[-\frac{1}{2},\frac{1}{2}\right]\mathrm{and}\beta_{k}∼\sqrt{6}\;\mathrm{Unif}\left[-\frac{1}{2},\frac{1}{2}\right]\mathrm{or}\epsilon_{k}∼G_{t=1}^{R}\mathrm{and}\beta_{k}∼G_{t=2}^{R}. \end{array}$$

These processes are implemented recursively; for technical details and background, see Appendix A.

**Proposition** **3.** *The discretization of Equation (72) can be re-expressed, up to order $\frac{1}{M}$ for M≫0, as follows:*

(73) $$\left(X_{M},N_{M}\right)∼G_{M}⊙\left(0,a\right),\mathrm{with}G_{M}=\left(\prod_{k=1}^{M}e^{\;\;\sum_{i=3}^{5}\sqrt{\frac{t\;D_{ii}}{M}}\epsilon_{k}^{i}A_{i}}\right)G_{0},$$

*with $\epsilon_{k}^{i}∼G_{t=1}^{R}$ stochastically independent normally distributed variables with $t=\frac{1}{2}\sigma^{2}=1$, and $D_{44}=D_{55}$.*

**Proof.**  In our construction, $\beta_{k}$ and $\gamma_{k}$ can be seen as the polar radius and the polar angle (on a periodic square [−π,π]×[−π,π]) of a Gaussian process with t=1 on a plane spanned by rotational generators $A_{4}$ and $A_{5}$. The key ingredient to obtain Equation (73) from Equation (72) is given by the following relation:

(75) $$e^{u\cos vA_{5}-u\sin vA_{4}}=e^{vA_{6}}e^{uA_{5}}e^{-vA_{6}},\text{for all }u,v\in R,$$

which we use for $u=\beta_{k}\sqrt{\frac{tD_{44}}{M}}$ and $v=\gamma_{k}\sqrt{\frac{tD_{44}}{M}}$.

The second ingredient is given by the Campbell–Baker–Hausdorff–Dynkin formula:

$$\mathrm{for}\mathrm{all}a_{i}=O\left(\frac{1}{\sqrt{M}}\right)\mathrm{and}\mathrm{for}M\mathrm{large},\mathrm{we}\mathrm{have}e^{a_{3}A_{3}}e^{a_{4}A_{4}}e^{a_{5}A_{5}}=e^{\left(a_{3}A_{3}+a_{4}A_{4}+a_{5}A_{5}\right)\left(1+O\left(\frac{1}{M}\right)\right)},$$

that allows to decompose the stochastic process in SE(3) into its spatial and angular parts. □

For the binning, we divide $R^{3}$ into cubes $c_{ijk}$, i,j,k∈Z, of size Δs×Δs×Δs:

(75) $$c_{ijk}:=\left[\left(i-\frac{1}{2}\right)\Delta s,\left(i+\frac{1}{2}\right)\Delta s\right]\times \left[\left(j-\frac{1}{2}\right)\Delta s,\left(j+\frac{1}{2}\right)\Delta s\right]\times \left[\left(k-\frac{1}{2}\right)\Delta s,\left(k+\frac{1}{2}\right)\Delta s\right].$$

We divide $S^{2}$ into bins $B_{l}$, l={1,⋯,b} for b∈N, with surface area $\sigma_{B_{l}}$ and maximal surface area $\sigma_{B}$. The number of random walks in a simulation with traveling time *t* that have their end point $x_{M}\in c_{ijk}$ with their orientation $n_{M}\in B_{l}$ is denoted with ${\#}_{t}^{ijkl}$. Furthermore, we define the indicator function

$$1_{c_{ijk},B_{l}}\left(x,n\right):=\left\{\begin{array}{cc} 1 & x\in c_{ijk},n\in B_{l},\\ 0 & \mathrm{otherwise}. \end{array}\right.$$

When the number of paths N→∞, the number of steps in each path M→∞ and the bin sizes tend to zero, the obtained distribution converges to the exact kernel:

(76) $$\begin{array}{c} \begin{array}{c} \lim_{N\to \infty }\lim_{\Delta s,\sigma_{B}\to 0}\lim_{M\to \infty }p_{t}^{\Delta s,\sigma_{B},N,M}\left(x,n\right)=K_{t}^{\alpha =1}\left(x,n\right),\\ \mathrm{with}p_{t}^{\Delta s,\sigma_{B},N,M}\left(x,n\right)=\sum_{l=1}^{b}\sum_{i,j,k\in Z}1_{c_{i,j,k},B_{l}}\left(x,n\right)\frac{{\#}_{t}^{ijkl}}{M{\left(\Delta s\right)}^{3}\sigma_{B_{l}}}. \end{array} \end{array}$$

**Figure 3:**
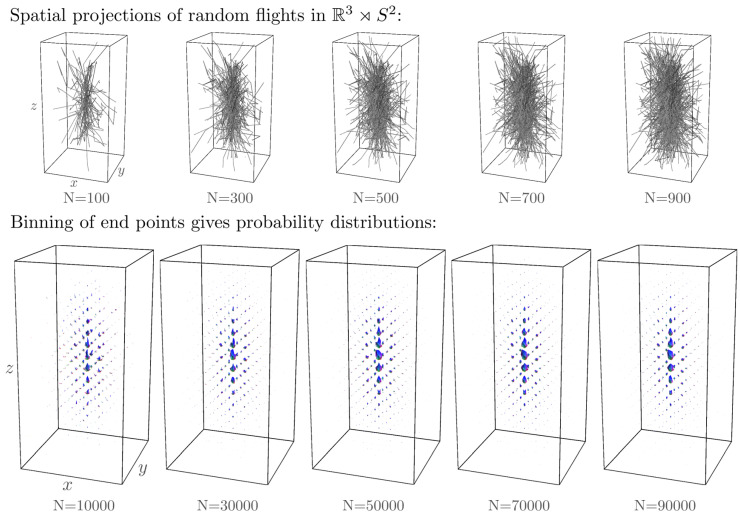
(**Top**) Spatial projections in $R^{3}$ of *N* sample paths of the discrete random walks (or rather “drunk man’s flights”) in $R^{3}⋊S^{2}$ for α=1, given by Equation (72), for increasing *N* (with $\sigma =\frac{4\pi }{252}$, Δs=1, M=40); and (**Bottom**) convergence of the Monte-Carlo simulation kernel in Equation (76) for α=1 and N→∞. As *N* increases, the Monte-Carlo simulation converges towards the exact solution. For a comparison of the exact diffusion kernel in Equation (70) and its Monte-Carlo approximation in Equation (76), see Figure 5.

The convergence is illustrated in Figure 3.

Monte-Carlo Simulation for α∈(0,1].

Let $q_{t,\alpha }:R^{+}\to R^{+}$ be the temporal probability density given by the inverse Laplace transform

(77) $$\begin{array}{c} q_{t,\alpha }\left(\tau \right)=L^{-1}\left(\lambda \to e^{-t\lambda^{\alpha }}\right)\left(\tau \right),\mathrm{with}\mathrm{in}\mathrm{particular}:\\ \;\mathrm{for}\alpha =\frac{1}{2}\mathrm{it}\mathrm{is}q_{t,\frac{1}{2}}\left(\tau \right)=\frac{t}{2\tau \sqrt{\pi \tau }}e^{-\frac{t^{2}}{4\tau }},\\ \;\mathrm{for}\alpha ↑1\mathrm{we}\mathrm{find}q_{t,\alpha }\left(\cdot \right)\to \delta_{t}\mathrm{in}\mathrm{distributional}\mathrm{sense}. \end{array}$$

For explicit formulas in the general case α∈(0,1], see [66]. Then, one can deduce from Theorem 3 that

(78) $$K_{t}^{\alpha }\left(x,n\right)=\int_{0}^{\infty }q_{t,\alpha }\left(\tau \right)\;K_{\tau }^{\alpha =1}\left(x,n\right)\;d\tau .$$

**Figure 4:**
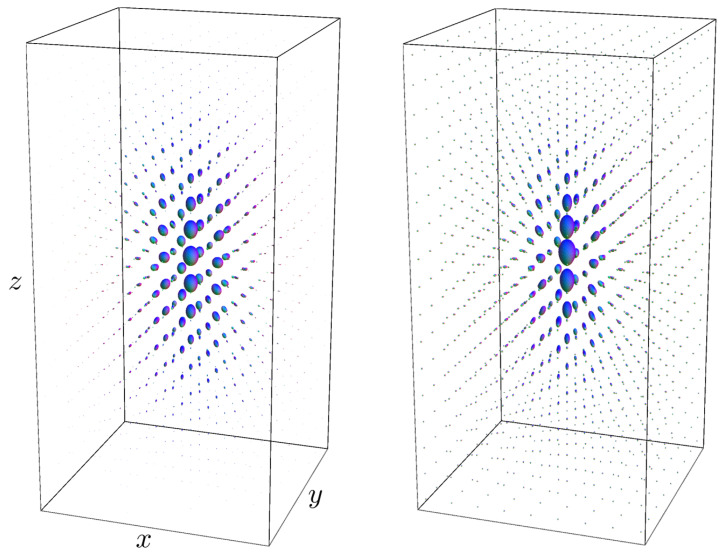
(**Left**) The degenerate diffusion kernel (Equation (70) for α=1 and t=2); and (**Right**) the degenerate Poisson kernel (Equation (70) for $\alpha =\frac{1}{2}$ and t=3.5). Parameters settings: $D_{44}\;=\;0.2,D_{33}\;=\;1,D_{11}=0$.

This allows us to directly use the Monte-Carlo simulations for the diffusion kernel α=1 for several time instances to compute a Monte-Carlo simulation of the α-stable Lévy kernels for α∈(0,1]. To this end, we replace the Monte Carlo approximation in Equation (76) for α=1 in the above Equation (79). See Figure 4, where we compare the diffusion kernel $K_{t}^{\alpha =1}$ to the Poisson kernel $K_{t}^{\alpha =\frac{1}{2}}$. See also Appendix A.2.1.

### 5.3. Comparison of Monte-Carlo Approximations of the Kernels to the Exact Solutions

In this section, we compute the probability density kernels $K_{t}^{\alpha }$ via the analytic approach of Section 5.1.2 (Equation (71), Theorem 3) and via the Monte-Carlo approximation of Section 5.2. The kernels are computed on a regular grid with each $\left(x_{i},y_{j},z_{k}\right)$ at the center of the cubes $c_{ijk}$ of Equation (75) with i,j=−3,⋯,3, k=−5,⋯,5, and Δs=0.5. The Monte-Carlo simulations also require spherical sampling which we did by a geodesic polyhedron that sub-divides each mesh triangle of an icosahedron into $n^{2}$ new triangles and projects the vertex points to the sphere. We set n=4 to obtain 252 (almost) uniformly sampled points on $S^{2}$.

The exact solution is computed using (truncated) spherical harmonics with l≤12. To obtain the kernel, we first solve the solution in the spatial Fourier domain and then do an inverse spatial Fast Fourier Transform. The resulting kernel $K_{t}^{\alpha }$ (where we literally follow Equation (71)) is only spatially sampled and provides for each $\left(x_{i},y_{j},z_{k}\right)$ an analytic spherical distribution expressed in spherical harmonics.

For the Monte-Carlo approximation, we follow the procedure described in Section 5.2. The kernel $K_{t}^{\alpha }$ is obtained by binning the end points of random paths on the quotient $R^{3}⋊S^{2}$ (cf. Equation (72)) and thereby approximate the limit in Equation (76). Each path is discretized with M=40 steps and in total $N=10^{10}$ random paths were generated. The sphere $S^{2}$ is divided into 252 bins with an average surface area of $\sigma_{B_{l}}\approx \frac{4\pi }{252}$.

**Figure 5:**
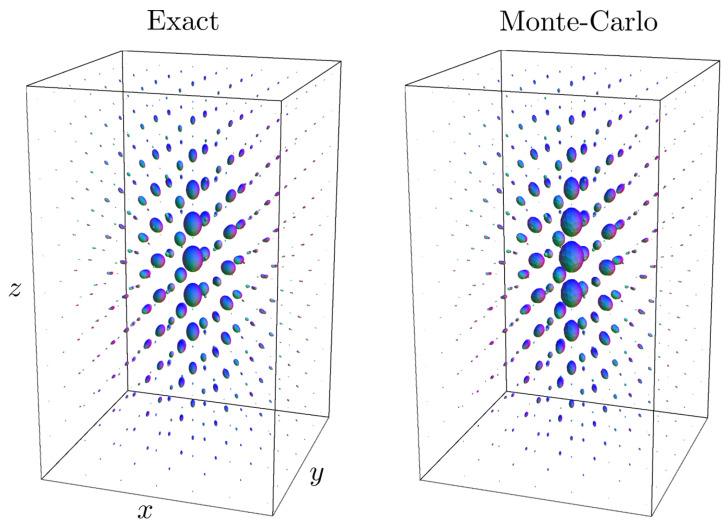
The exact kernel $K_{t}^{\alpha }$ and its Monte-Carlo approximation for t=2, α=1, $D_{33}=1$, $D_{44}=0.2$.

In Figure 1 and Figure 3, Figure 4 and Figure 5, we set $D_{33}=1$, $D_{44}=0.2$. In the comparison between the kernels $K_{t}^{\alpha =1}$ with $K_{t}^{\alpha =0.5}$, we set t=2 and t=3.5, respectively, to match the full width at half maximum value of the distributions. In Figure 1, Figure 3 and Figure 5, we set α=1 and t=2. In Figure 1, Figure 3 and Figure 4, we sample the grid in Equation (75) with |i|,|j|≤4, |k|≤8.

Figure 5 shows that the Monte-Carlo kernel closely approximates the exact solution and since the exact solutions can be computed at arbitrary spherical resolution, it provides a reliable way to validate numerical methods for α-stable Lévy processes on $R^{3}⋊S^{2}$.

## 6. Conclusions

We set up a Fourier transform $F_{G/H}$ on the homogeneous space of positions and orientations. The considered Fourier transform acts on functions that are bi-invariant with respect to the action of subgroup *H*. We provide explicit formulas (relative to a basis of modified spherical harmonics) for the transform, its inverse, and its Plancherel formula, in Theorem 1.

Then, we use this Fourier transform to derive new exact solutions to the probability kernels of α-stable Lévy processes on G/H, including the diffusion PDE for Wiener processes, which is the special case α=1. They are obtained by spectral decomposition of the evolution operator in Theorem 2.

New formulas for the probability kernels are presented in Theorem 3. There, the general case 0<α<1 follows from the case α=1 by taking the fractional power of the eigenvalues. In comparison to previous formulas in [40] for the special case α=1 obtained via a spatial Fourier transform, we have more concise formulas with a more structured evolution operator in the Fourier domain of G/H, where we rely on ordinary spherical harmonics, and where we reduce the dimension of the manifold over which it is integrated from 3 to 1 (as can be seen in Theorem 3).

We introduce stochastic differential equations (or rather stochastic integral equations) for the α-stable Lévy processes in Appendix A.1, and we provide simple discrete approximations where we rely on matrix exponentials in the Lie group SE(3) in Proposition 3.

We verified the exact solutions and the stochastic process formulations, by Monte-Carlo simulations that confirmed to give the same kernels, as shown in Figure 5. We also observed the expected behavior that the probability kernels for 0<α<1 have heavier tails, as shown in Figure 4.

The PDEs and the probability kernels have a wide variety of applications in image analysis (crossing-preserving, contextual enhancement of diffusion-weighted MRI, cf. [45,46,49,94,97,98] or in crossing-preserving diffusions in 3D scalar images [18]), robotics [4,5,57] and probability theory [56,61]. The generalizations to α∈(0,1] allow for longer range interactions between local orientations (due to the heavy tails). This is also of interest in machine learning, where convolutional neural networks on the homogeneous space of positions and orientations [9,12] can be extended to 3D [67,68], which may benefit from the PDE descriptors and the Fourier transform presented here.

## Abbreviations

The following abbreviations and symbols are used in this manuscript:

UIR	Unitary Irreducible Representation	
*G*	The rigid body motions group SE(3)	Equation (1)
a	The reference axis $a=e_{z}={\left(0,0,1\right)}^{T}$	Equation (3)
*H*	The subgroup that stabilizes (0,a)	Equation (3)
G/H	The homogeneous space of positions and orientations $R^{3}⋊S^{2}$	Equation (3)
$\bar{U}$	The spatial Fourier transform of *U*	Equation (18)
$\hat{U}$	The Fourier transform $\hat{U}=F_{G/H}U$	Equation (43)
α	Parameter of the α-Stable processes (indexing fractional power of the generator)	Equation (10)

$\bar{\alpha }$	Rotation angle around reference axis $a=e_{z}=\left(0,0,1\right)$	Remark 7
$\sigma^{p,s}$	UIR of G=SE(3)	Equation (32)
${\bar{\sigma }}^{p,s}$	the action on the quotient corresponding to $\sigma^{p,s}$	Definition 7
${\tilde{K}}_{t}^{\alpha }$	The probability kernel on *G*	Equation (26)
$K_{t}^{\alpha }$	The probability kernel on G/H	Equation (27)
${\tilde{W}}_{\alpha }$	Solution of the PDE on *G*	Equation (10)
$W_{\alpha }$	Solution of the PDE on G/H	Equation (6)
${\tilde{Q}}_{\alpha }$	Evolution generator of the PDE on *G*	Equation (11)
$Q_{\alpha }$	Evolution generator of the PDE on G/H	Equation (7)
$R_{n}$	Any rotation that maps a onto n	Remark 2
$R_{v,\phi }$	A counter-clockwise rotation about axis v with angle ϕ	Remark 2
$P_{t}$	Lévy Processes on G/H	Definition A1
${\bar{P}}_{t}$	Lévy Processes on $R^{3}\times R^{3}$	Equation (A4)
$q_{t,\alpha }$	The kernel relating $K_{t}^{\alpha }$ and $K_{t}^{1}$	Equation (77)
$Y^{l,m}$	The ordinary spherical harmonics	Proposition 2
$Y_{s}^{l,m}$	The modified spherical harmonics according to [4]	Proposition 2
$Y_{\omega }^{l,m}$	The generalized spherical harmonics according to [40]	Definition 10
$\Phi_{\omega }^{l,m}$	The spheroidal wave basis function for $L_{2}\left(S^{2}\right)$	Definition 11
$\left(\bar{\alpha },\beta ,\gamma \right)$	ZYZ Euler angles.	Equation (A12)

## Appendix A. Probability Theory

### Appendix A.1. Lévy Processes on R 3 ⋊S 2

In the next definition, we define Lévy processes on our manifold of interest $G/H=R^{3}⋊S^{2}$. Recall, that the action of G=SE(3) on G/H is given by Equation (4). As a prerequisite, we define the “difference” of two random variables $P_{1}=\left(X_{1},N_{1}\right)$ and $P_{2}=\left(X_{2},N_{2}\right)$ in $R^{3}⋊S^{2}$:

(A1) $$G_{2}^{-1}⊙P_{1}={\left(X_{2},R_{N_{2}}\right)}^{-1}⊙\left(X_{1},N_{1}\right)=\left(R_{N_{2}}^{T}\left(X_{1}-X_{2}\right),R_{N_{2}}^{T}N_{1}\right),$$

where we relate random variables on G/H and in *G* via P=G⊙(0,a), according to Equation (39).

We assume that $P_{1}$ and $P_{2}$ are chosen such that the distribution of $G_{2}^{-1}⊙P_{1}$ is invariant under the choice of rotation variable $R_{N_{2}}\in SO\left(3\right)$, which maps reference axis a onto $N_{2}$. This is done in view of the homogeneous space structure in Equation (3) and the fact that Lévy processes on Lie groups such as G=SE(3) require Lie group inversion in their definition (see, e.g., [99]).

**Definition** **A1.**
*A stochastic process $\left\{P_{t}\;:\;t\geq 0\right\}$ on G/H is a Lévy process if the following conditions hold:*

1. *For any n≥1 and $0\;\leq \;t_{0}<\;t_{1}\;<\;\ldots \;<\;t_{n}$, the variables $P_{t_{0}}$, $G_{t_{0}}^{-1}⊙P_{t_{1}}$, …, $G_{t_{n-1}}^{-1}⊙P_{t_{n}}$ are independent.*
2. *The distribution of $G_{s}^{-1}⊙P_{s+t}$ does not depend on s≥0.*
3. *$P_{0}=\left(0,a\right)$ almost surely.*
4. *It is stochastically continuous, i.e., $\lim_{s\to t}P\left[d\left(P_{s},P_{t}\right)>\varepsilon \right]=0$, ∀ε>0.*
  *Here, $d\left(\left(x_{1},n_{1}\right),\left(x_{2},n_{2}\right)\right)={\left|x_{1}-x_{2}\right|}^{2}+\arccos^{2}\left(n_{1}\cdot n_{2}\right)$.*

Let us consider the solutions

$$W_{\alpha }\left(x,n,t\right)=\left(K_{t}^{\alpha }*U\right)\left(x,n\right)$$

of our linear PDEs of interest in Equation (6) for α∈(0,1] fixed. Let us consider the case where $U∼\delta_{\left(0,a\right)}$, so that the solutions are the probability kernels $K_{t}^{\alpha }$ themselves. We consider the random variables $P_{t}^{\alpha }$ such that their probability densities are given by

(A2) $$P\left(P_{t}^{\alpha }=\left(x,n\right)\right)=K_{t}^{\alpha }\left(x,n\right)\mathrm{for}\mathrm{all}t\geq 0,\left(x,n\right)\in R^{3}⋊S^{2}.$$

**Proposition** **A1.**
*The stochastic process $\left\{P_{t}^{\alpha }\;:\;t\geq 0\right\}$ is a Lévy processes on $R^{3}⋊S^{2}$.*

**Proof.** We first address Items 1 and 2. On G=SE(3), one has for two stochastically independent variables:

$$P\left(G_{1}G_{2}=g\right)=\int_{G}P\left(G_{2}=h^{-1}g\right)P\left(G_{1}=h\right)\;dh.$$

In particular, for $G_{1}=G_{t}∼{\tilde{K}}_{t}^{\alpha }$ and $G_{2}=G_{s}∼{\tilde{K}}_{s}^{\alpha }$, we have

$$G_{s}G_{t}∼{\tilde{K}}_{t}^{\alpha }*{\tilde{K}}_{s}^{\alpha }={\tilde{K}}_{t+s}^{\alpha }\;\mathrm{and}\;G_{s}^{-1}G_{t+s}=G_{t}∼{\tilde{K}}_{t}^{\alpha },$$

which is due to $e^{t{\tilde{Q}}_{\alpha }}\circ e^{s{\tilde{Q}}_{\alpha }}=e^{\left(t+s\right){\tilde{Q}}_{\alpha }}$ (recall Equation (55)). Similarly, on the quotient G/H, we have

$$G_{s}^{-1}⊙P_{s+t}=P_{t}∼K_{t}^{\alpha }.$$

Furthermore, the choice of $G_{s}$ such that $G_{s}⊙\left(0,a\right)=\left(0,a\right)$ does not matter, since

$$P\left({\left(0,R_{a,\bar{\alpha }}\right)}^{-1}G_{s}^{-1}⊙P_{s+t}=\left(x,n\right)\right)=K_{t}^{\alpha }\left(\left(0,R_{a,\bar{\alpha }}\right)⊙\left(x,n\right)\right)=K_{t}^{\alpha }\left(x,n\right)$$

(recall Equation (30)). Item 3 is obvious since we have $P_{0}=\delta_{\left(0,a\right)}$. Item 4 follows by strong continuity of the semigroup operators ([64], Thm. 2), [66]. □

**Lemma** **A1.**
*The kernels $K_{t}^{\alpha }$ are infinitely divisible, i.e.*

$$K_{t}^{\alpha }*K_{s}^{\alpha }=K_{t+s}^{\alpha }\;\text{for all }s,t\geq 0.$$

**Proof.** The infinite divisibility directly follows from Corollary 1 and $F_{G/H}\left(K_{t}^{\alpha }*K_{s}^{\alpha }\right)=F_{G/H}\left(K_{t}^{\alpha }\right)\circ F_{G/H}\left(K_{t}^{\alpha }\right)=F_{G/H}\left(K_{t+s}^{\alpha }\right)$, which is clear due to Equation (70).

**Remark** **A1.**
*Recall that on $R^{n}$ a Lévy process $X_{t}$ is called α-stable if*

(A3) $$a^{-\frac{1}{2\alpha }}X_{at}∼X_{t}\;\text{for all }a>0.$$

*This convention and property applies to all n∈N, cf. [61]. Next, we come to a generalization of α-stability but then for the processes $P_{t}$. Here, an embedding of $R^{3}⋊S^{2}$ into $R^{6}=R^{3}\times R^{3}$ is required to give a meaning to α-stability and a scaling relation on $P_{t}=\left(X_{t},N_{t}\right)$ that is similar to Equation (A3).*

### Appendix A.2. SDE Formulation of α-Stable Lévy Processes on $R^{3}⋊S^{2}$

Consider the Lévy processes $\left\{P_{t}\;:\;t\geq 0\right\}$ on $R^{3}⋊S^{2}$ given by Equation (A2). They give rise to the Forward Kolmogorov PDEs in Equation (6) in terms of their stochastic differential equation (SDE) according to the book of Hsu on Stochastic Analysis on Manifolds [60].

We apply ([60], Prop.1.2.4) on the embedding map $\Phi :R^{3}\times R^{3}\to R^{3}⋊S^{2}$ given by

$$\Phi :\left(x,\bar{n}\right)\mapsto \Phi \left(x,\bar{n}\right)=\left(x,\frac{\bar{n}}{∥\bar{n}∥}\right)=\left(x,n\right).$$

Note that $\Phi_{*}=D\Phi =\left(I,\frac{1}{∥\bar{n}∥}\left(I-\frac{\bar{n}}{∥\bar{n}∥}⊗\frac{\bar{n}}{∥\bar{n}∥}\right)\right)$. Here, *I* denotes the identity map on $R^{3}$.

Let us first concentrate on α=1. In this case, our PDE in Equation (6) becomes a diffusion PDE that is the forward Kolmogorov equation of a Wiener process $P_{t}=\left(X_{t},N_{t}\right)$ on $R^{3}⋊S^{2}$. Next, we relate this Wiener process to a Wiener process $\left(W_{t}^{\left(1\right)},{\bar{W}}_{t}^{\left(2\right)}\right)$ in the embedding space $R^{3}\times R^{3}$. We write down the stochastic differential equation (SDE) and show that Equation (72) boils down to discretization of the stochastic integral (in Îto sense) solving the SDE.

Next, we define ${\bar{P}}_{t}=\left(X_{t},{\bar{N}}_{t}\right)$ by the SDE in the embedding space:

(A4) $$d{\bar{P}}_{t}=\bar{s}{|}_{{\bar{P}}_{t}}\circ dW_{t},$$

where $W_{t}=\left(W_{t}^{\left(1\right)},{\bar{W}}_{t}^{\left(2\right)}\right)$, with $W_{t}^{\left(1\right)}$ and ${\bar{W}}_{t}^{\left(2\right)}$ being Wiener processes in $R^{3}$; and where

$$\bar{s}{|}_{\bar{P}}\left(dx,d\bar{n}\right)=\left(\begin{array}{c} {\bar{s}}^{\left(1\right)}{|}_{\bar{P}}\left(dx,d\bar{n}\right)\\ {\bar{s}}^{\left(2\right)}{|}_{\bar{P}}\left(dx,d\bar{n}\right) \end{array}\right)=\left(\begin{array}{c} \sqrt{D_{33}}\;\frac{\bar{N}}{∥\bar{N}∥}\;\;\left(\frac{\bar{N}}{∥\bar{N}∥}\cdot dx\right)\\ \sqrt{D_{44}}\;d\bar{n} \end{array}\right).$$

Here, Index (1) stands for the spatial part and Index (2) stands for the angular part.

Now, we define a corresponding process on $R^{3}⋊S^{2}$:

$$P_{t}=\Phi \left({\bar{P}}_{t}\right).$$

Then, the SDE for $P_{t}=\left(X_{t},N_{t}\right)$ becomes (see ([60], Prop.1.2.4))

$$dP_{t}=d\left(\Phi \circ {\bar{P}}_{t}\right)\Leftrightarrow \left\{\begin{array}{c} dX_{t}={\left.{\bar{s}}^{\left(1\right)}\right|}_{{\bar{P}}_{t}}\circ dW_{t}^{\left(1\right)},\\ dN_{t}=P_{{\langle N_{t}\rangle }^{⊥}}{\left.{\bar{s}}^{\left(2\right)}\right|}_{{\bar{P}}_{t}}\circ d{\bar{W}}_{t}^{\left(2\right)}, \end{array}\right.$$

where $N_{t}=\frac{{\bar{N}}_{t}}{∥{\bar{N}}_{t}∥}$; and where $P_{{\langle N_{t}\rangle }^{⊥}}=\left(I-N_{t}⊗N_{t}\right)$ denotes the orthogonal projection to the tangent plane perpendicular to $N_{t}$.

Therefore, we have the following SDE on $R^{3}⋊S^{2}$:

(A5) $$\left\{\begin{array}{c} dX_{t}=\sqrt{D_{33}}\;N_{t}\left(N_{t}\cdot dW_{t}^{\left(1\right)}\right),\\ dN_{t}=\sqrt{D_{44}}\;P_{{\langle N_{t}\rangle }^{⊥}}d{\bar{W}}_{t}^{\left(2\right)} \end{array}\right.$$

Thus, integrating the SDE, we obtain the following stochastic integral (in Îto form):

(A6) $$\left\{\begin{array}{c} X_{t}=X_{0}+\sqrt{D_{33}}\int_{0}^{t}N_{s}\left(N_{s}\cdot dW_{s}^{\left(1\right)}\right)=X_{0}+\sqrt{D_{33}}\underset{M\to \infty }{ms-lim}\sum_{k=1}^{M}N_{t_{k-1}}\left(N_{t_{k-1}}\cdot \left(W_{t_{k}}^{\left(1\right)}-W_{t_{k-1}}^{\left(1\right)}\right)\right),\\ N_{t}=\underset{M\to \infty }{ms-lim}\prod_{k=1}^{M}\exp_{S^{2}}\left(\sqrt{D_{44}}\left(I-N_{t_{k-1}}⊗N_{t_{k-1}}\right)\left({\bar{W}}_{t_{k}}^{\left(2\right)}-{\bar{W}}_{t_{k-1}}^{\left(2\right)}\right)\right)N_{0}. \end{array}\right.$$

Here, $\exp_{S^{2}}\left(V\right)n_{0}$ denotes the exponential map on a sphere, i.e., its value is the end point (for t=1) of a geodesic starting from $n_{0}\in S^{2}$ with the tangent vector $V\in T_{n_{0}}S^{2}$. Note that, in the formula above, the symbol ∏ denotes the composition

$$\prod_{k=1}^{M}\exp_{S^{2}}\left(V_{k}\right)n_{0}=\left(\exp_{S^{2}}\left(V_{M}\right)\circ \ldots \circ \exp_{S^{2}}\left(V_{1}\right)\right)n_{0}.$$

Note that $\sqrt{D_{33}}\left(W_{t_{k}}^{\left(1\right)}-W_{t_{k-1}}^{\left(1\right)}\right)=\sqrt{D_{33}}W_{t_{k}-t_{k-1}}^{\left(1\right)}=\sqrt{\frac{tD_{33}}{M}}\epsilon_{k}$, where $\epsilon_{k}∼W_{1}^{\left(1\right)}$, i.e., $\epsilon_{k}∼G_{t=1}$.

For M∈N fixed, we propose a discrete approximation for the stochastic integrals in Equation (A6):

(A7) $$\left\{\begin{array}{c} X_{M}=X_{0}+\sum_{k=1}^{M}\sqrt{\frac{tD_{33}}{M}}\epsilon_{k}N_{k-1},\\ N_{M}=\left(\prod_{k=1}^{M}R_{a,\gamma_{k}}R_{e_{y},\beta_{k}\sqrt{\frac{tD_{44}}{M}}}\right)N_{0}, \end{array}\right.$$

with $\epsilon_{k}∼G_{t=1}^{R}∼N\left(0,\sigma =\sqrt{2}\right)$ stochastically independent Gaussian distributed on R with t=1; with uniformly distributed $\gamma_{k}∼\mathrm{Unif}\left(R/(2\pi Z)\equiv [-\pi ,\pi )\right)$; and with $\beta_{k}∼g$, where $g:R\to R^{+}$ equals $g\left(\beta \right)=\frac{\left|\beta \right|}{2}\;e^{-\frac{\beta^{2}}{4}}$. The choice of *g* is done by application of the theory of isotropic stochastic processes on Riemannian manifolds by Pinsky [96], where we note that

$$G_{t}^{R^{2}}\left(\beta \cos\gamma ,\beta \sin\gamma \right)=g\left(\beta \right)\;\mathrm{Unif}\left([-\pi ,\pi )\right)\left(\gamma \right),\;\beta \in R,\;\gamma \in \left[-\pi ,\pi \right).$$

Now, in the numerical simulation, we can replace *g* by $G_{t=2}^{R}$ due to the central limit theorem on R and

$$\mathrm{Var}\left(\beta \right)=\int_{-\infty }^{\infty }\beta^{2}g\left(\beta \right)d\beta =2\int_{0}^{\infty }\beta^{2}g\left(\beta \right)d\beta =2.$$

#### Appendix A.2.1. From the Diffusion Case α = 1 to the General Case α ∈ (0,1]

For the case α∈(0,1], we define the (fractional) random processes by their probability densities

(A8) $$\begin{array}{c} P\left(P_{t}^{\alpha }=\left(x,n\right)\right)=\int_{0}^{\infty }q_{t,\alpha }\left(\tau \right)\;P\left(P_{\tau }=\left(x,n\right)\right)\;d\tau ,\\ P\left({\bar{P}}_{t}^{\alpha }=\left(x,\bar{n}\right)\right)=\int_{0}^{\infty }q_{t,\alpha }\left(\tau \right)\;P\left({\bar{P}}_{\tau }=\left(x,\bar{n}\right)\right)\;d\tau . \end{array}$$

Recal that the kernel $q_{t,\alpha }\left(\tau \right)$ is given by Equation (77). For Monte-Carlo simulations, one can use Equation (78), or alternatively use $P_{t_{M}}^{\alpha }\approx \prod_{i=1}^{M}G_{T_{i}}⊙P_{0},\mathrm{for}M\gg 0$, where $P_{0}$ is almost surely (0,a), with $T_{i}$ a temporal random variable with $P\left(T_{i}=\tau \right)=q_{t_{i},\alpha }\left(\tau \right)$, with $t_{i}=\frac{i}{M}t$ and $G_{t_{i}}$ given by Equation (73).

#### Appendix A.2.2. α-Stability of the Lévy Process

Due to the absence of suitable dilations on G/H, we resort to the embedding space where α-stability is defined. The Lévy process $\left\{{\bar{P}}_{t}^{\alpha }=\left(X_{t}^{\alpha },{\bar{N}}_{t}^{\alpha }\right)\;|\;t\geq 0\right\}$ associated to the Lévy process $\left\{P_{t}^{\alpha }=\left(X_{t}^{\alpha },N_{t}^{\alpha }\right)\;|\;t\geq 0\right\}$ in $R^{3}⋊S^{2}$ is α-stable, i.e., for all a,t>0 we have (by Equations (A5) and (78))

$$\begin{array}{c} a^{-\frac{1}{2\alpha }}X_{at}^{\alpha }∼X_{t}^{\alpha }\mathrm{and}\;a^{-\frac{1}{2\alpha }}{\bar{N}}_{at}^{\alpha }∼{\bar{N}}_{t}^{\alpha }. \end{array}$$

## Appendix B. Left-Invariant Vector Fields on SE(3) via Two Charts

We need two charts to cover SO(3). When using the following coordinates (ZYZ-Euler angles) for $SE\left(3\right)=R^{3}⋊SO\left(3\right)$ for the first chart:

(A9) $$g=\left(x,y,z,R_{e_{z},\gamma }R_{e_{y},\beta }R_{e_{z},\bar{\alpha }}\right),\mathrm{with}\beta \in \left(0,\pi \right),\bar{\alpha },\gamma \in \left[0,2\pi \right),$$

Equation (9) yields the following formulas for the left-invariant vector fields:

(A10) $$\begin{array}{c} \;\;A_{1}{|}_{g}=\left(\cos\bar{\alpha }\cos\beta \cos\gamma -\sin\bar{\alpha }\sin\gamma \right)\partial_{x}+\left(\sin\bar{\alpha }\cos\gamma +\cos\bar{\alpha }\cos\beta \sin\gamma \right)\partial_{y}-\cos\bar{\alpha }\sin\beta \;\partial_{z}\\ \;\;A_{2}{|}_{g}=\left(-\sin\bar{\alpha }\cos\beta \cos\gamma -\cos\bar{\alpha }\sin\gamma \right)\partial_{x}+\left(\cos\bar{\alpha }\cos\gamma -\sin\bar{\alpha }\cos\beta \sin\gamma \right)\partial_{y}+\sin\bar{\alpha }\sin\beta \;\partial_{z}\\ \;\;A_{3}{|}_{g}=\sin\beta \cos\gamma \;\partial_{x}+\sin\beta \sin\gamma \;\partial_{y}+\cos\beta \;\partial_{z},\\ \;\;A_{4}{|}_{g}=\;\cos\bar{\alpha }\cot\beta \;\partial_{\bar{\alpha }}+\sin\bar{\alpha }\;\partial_{\beta }-\frac{\cos\bar{\alpha }}{\sin\beta }\;\partial_{\gamma },\\ \;\;A_{5}{|}_{g}=-\sin\bar{\alpha }\cot\beta \;\partial_{\bar{\alpha }}+\cos\bar{\alpha }\;\partial_{\beta }+\frac{\sin\bar{\alpha }}{\sin\beta }\;\partial_{\gamma },\\ \;\;A_{6}{|}_{g}=\partial_{\bar{\alpha }}. \end{array}$$

We observe that

(A11) $${\underline{A}}_{gh_{\bar{\alpha }}}\equiv {\left(R_{e_{z},\bar{\alpha }}⊕R_{e_{z},\bar{\alpha }}\right)}^{T}{\underline{A}}_{g},\;\mathrm{where}{\underline{A}}_{g}=\left(A_{1}{{|}_{g},\ldots ,A_{6}|}_{g}\right).$$

The above formulas do not hold for β=π or β=0. Thus, we even lack expressions for our left-invariant vector fields at the unity element (0,I)∈SE(3) when using the standard ZYZ-Euler angles. Therefore, one formally needs a second chart, for example the XYZ-coordinates in [84,87,100]:

(A12) $$g=\left(x,y,z,R_{e_{x},\tilde{\gamma }}R_{e_{y},\tilde{\beta }}R_{e_{z},\bar{\alpha }}\right),\;\mathrm{with}\tilde{\beta }\in \left[-\pi ,\pi \right),\bar{\alpha }\in \left[0,2\pi \right),\;\tilde{\gamma }\in \left(-\pi /2,\pi /2\right),$$

Equation (9) yields the following formulas for the left-invariant vector fields (only for $|\tilde{\beta }|\neq \frac{\pi }{2}$):

(A13) $$\begin{array}{c} \;\;A_{1}{|}_{g}=\cos\bar{\alpha }\cos\tilde{\beta }\;\partial_{x}+\left(\cos\tilde{\gamma }\sin\bar{\alpha }+\cos\bar{\alpha }\sin\tilde{\beta }\sin\tilde{\gamma }\right)\;\partial_{y}+\left(\sin\bar{\alpha }\sin\tilde{\gamma }-\cos\bar{\alpha }\sin\tilde{\beta }\cos\tilde{\gamma }\right)\;\partial_{z}\\ \;\;A_{2}{|}_{g}=-\sin\bar{\alpha }\cos\tilde{\beta }\;\partial_{x}+\left(\cos\bar{\alpha }\cos\tilde{\gamma }-\sin\bar{\alpha }\sin\tilde{\beta }\sin\tilde{\gamma }\right)\partial_{y}+\left(\sin\bar{\alpha }\sin\tilde{\beta }\cos\tilde{\gamma }+\cos\bar{\alpha }\sin\tilde{\gamma }\right)\;\partial_{z}\\ \;\;A_{3}{|}_{g}=\sin\tilde{\beta }\;\partial_{x}-\cos\tilde{\beta }\sin\tilde{\gamma }\;\partial_{y}+\cos\tilde{\beta }\cos\tilde{\gamma }\;\partial_{z},\\ \;\;A_{4}{|}_{g}=-\cos\bar{\alpha }\tan\tilde{\beta }\;\partial_{\bar{\alpha }}+\sin\bar{\alpha }\;\partial_{\tilde{\beta }}+\frac{\cos\bar{\alpha }}{\cos\tilde{\beta }}\partial_{\tilde{\gamma }},\\ \;\;A_{5}{|}_{g}=\sin\bar{\alpha }\tan\tilde{\beta }\;\partial_{\bar{\alpha }}+\cos\bar{\alpha }\partial_{\tilde{\beta }}-\frac{\sin\bar{\alpha }}{\cos\beta }\;\partial_{\tilde{\gamma }},\\ \;\;A_{6}{|}_{g}=\partial_{\bar{\alpha }}. \end{array}$$

## References

[1] Zettl A. (2005). Sturm-Liouville Theory.

[2] Kato T. (1976). Operators in Hilbert spaces. Perturbation Theory for Linear Operators.

[3] Rudin W. (1991). Functional Analysis.

[4] Chirikjian G.S., Kyatkin A.B. (2000). Engineering Applications of Noncommutative Harmonic Analysis: With Emphasis on Rotation and Motion Groups.

[5] Chirikjian G.S. (2011). Stochastic Models, Information Theory, and Lie Groups: Analytic Methods and Modern Applications.

[6] Saccon A., Aguiar A.P., Hausler A.J., Hauser J., Pascoal A.M. Constrained motion planning for multiple vehicles on SE(3). Proceedings of the 2012 IEEE 51st IEEE Conference on Decision and Control (CDC).

[7] Henk Nijmeijer A.V.D.S. (1990). Nonlinear Dynamical Control Systems.

[8] Ali S., Antoine J., Gazeau J. (1999). Coherent States, Wavelets and Their Generalizations.

[9] Bekkers E., Lafarge M., Veta M., Eppenhof K., Pluim J., Duits R. (2018). Roto-Translation Covariant Convolutional Networks for Medical Image Analysis. Medical Image Computing and Computer Assisted Intervention—MICCAI 2018.

[10] Bekkers E., Loog M., ter Haar Romeny B., Duits R. (2017). Template matching via densities on the roto-translation group. IEEE Trans. Pattern Anal. Mach. Intell..

[11] Cohen T.S., Geiger M., Weiler M. (2018). Intertwiners between Induced Representations (with Applications to the Theory of Equivariant Neural Networks). arXiv.

[12] Cohen T., Welling M. Group equivariant convolutional networks. Proceedings of the International Conference on Machine Learning.

[13] Sifre L., Mallat S. Rotation, scaling and deformation invariant scattering for texture discrimination. Proceedings of the 2013 IEEE Conference on Computer Vision and Pattern Recognition.

[14] Duits R., Felsberg M., Granlund G., ter Haar Romeny B. (2006). Image Analysis and Reconstruction using a Wavelet Transform Constructed from a Reducible Representation of the Euclidean Motion Group. Int. J. Comput. Vis..

[15] Citti G., Sarti A. (2006). A Cortical Based Model of Perceptual Completion in the Roto-Translation Space. J. Math. Imaging Vis..

[16] Duits R., Fuehr H., Janssen B., Florack L., van Assen H. (2013). Evolution equations on Gabor transforms and their applications. ACHA.

[17] Prandi D., Gauthier J.P. (2018). A Semidiscrete Version of the Citti-Petitot-Sarti Model as a Plausible Model for Anthropomorphic Image Reconstruction and Pattern Recognition.

[18] Janssen M.H.J., Janssen A.J.E.M., Bekkers E.J., Bescós J.O., Duits R. (2018). Design and Processing of Invertible Orientation Scores of 3D Images. J. Math. Imaging Vis..

[19] Boscain U., Duplaix J., Gauthier J., Rossi F. (2012). Anthropomorphic Image Reconstruction via Hypoelliptic Diffusion. SIAM J. Control Optim..

[20] Schur I. (1968). Vorlesungen über Invariantentheorie.

[21] Dieudonné J. (1977). Treatise on Analysis.

[22] Folland G.B. (1994). A Course in Abstract Harmonic Analysis.

[23] Agrachev A., Boscain U., Gauthier J.P., Rossi F. (2009). The intrinsic hypoelliptic Laplacian and its heat kernel on unimodular Lie groups. J. Funct. Anal..

[24] Führ H. (2005). Abstract Harmonic Analysis of Continuous Wavelet Transforms.

[25] Mackey G.W. (1949). Imprimitivity for Representations of Locally Compact Groups I. Proc. Natl. Acad. Sci. USA.

[26] Sugiura M. (1990). Unitary Representations and Harmonic Analysis: An Introduction.

[27] Dixmier J. (1981). C^*^-algebras.

[28] Ghaani Farashani A. (2017). Operator-valued Fourier transforms over homogeneous spaces of compact groups. Groups Geom. Dyn..

[29] Ghaani Farashani A. (2017). Poisson summation formulas over homogeneous spaces of compact groups. Anal. Math. Phys..

[30] Ghaani Farashani A. (2017). Plancherel (trace) formulas over homogeneous spaces of compact groups. Can. Math. Bull..

[31] Ghaani Farashahi A. (2017). Relative Fourier transforms over canonical homogeneous spaces of semi-direct product groups with abelian normal factor. J. Korean Math. Soc..

[32] Gaveau B. (1977). Principe de moindre action, propagation de la chaleur et estimees sous elliptiques sur certains groupes nilpotents. Acta Math..

[33] Duits R., van Almsick M. (2008). The explicit solutions of linear left-invariant second order stochastic evolution equations on the 2D Euclidean motion group. Q. Appl. Math..

[34] Duits R., Franken E. (2009). Line Enhancement and Completion via Linear Left Invariant Scale Spaces on SE(2).

[35] Duits R., van Almsick M. (2005). The Explicit Solutions of Linear Left-Invariant Second Order Stochastic Evolution Equations on the 2D-Euclidean Motion Group.

[36] Duits R., Franken E. (2010). Left-invariant parabolic evolutions on SE(2) and contour enhancement via invertible orientation scores Part II: Nonlinear left-invariant diffusions on invertible orientation scores. Q. Appl. Math..

[37] Zhang J., Duits R., Sanguinetti G., ter Haar Romeny B.M. (2016). Numerical Approaches for Linear Left-invariant Diffusions on SE(2), their Comparison to Exact Solutions, and their Applications in Retinal Imaging. Numer. Methods Theory Appl..

[38] Mumford D. (1994). Elastica and Computer Vision. Algebraic Geometry and its Applications.

[39] Petitot J. (2003). The neurogeometry of pinwheels as a sub-Riemannian contact structure. J. Physiol. Paris.

[40] Portegies J.M., Duits R. (2017). New exact and numerical solutions of the (convection–)diffusion kernels on SE(3). Differ. Geom. Appl..

[41] Chirikjian G., Albeverio S., Cruzeiro A., Holm D. (2017). Degenerate Diffusions and Harmonic Analysis on SE(3): A Tutorial. Stochastic Geometric Mechanics.

[42] Park W., Liu Y., Zhou Y., Moses M., Chirikjian G. (2008). Kinematic State Estimation and Motion Planning for Stochastic Nonholonomic Systems Using the Exponential Map. Robotica.

[43] Chirikjian G., Wang Y. (2000). Conformational Statistics of Stiff Macromolecules as Solutions to PDEs on the Rotation and Motion Groups. Phys. Rev. E.

[44] Portegies J. (2018). PDEs on the Lie Group SE(3). Ph.D. Thesis.

[45] Portegies J.M., Fick R.H.J., Sanguinetti G.R., Meesters S.P.L., Girard G., Duits R. (2015). Improving Fiber Alignment in HARDI by Combining Contextual PDE Flow with Constrained Spherical Deconvolution. PLoS ONE.

[46] Momayyez-Siahkal P., Siddiqi K. 3D Stochastic Completion Fields for Fiber Tractography. Proceedings of the 2009 IEEE Computer Society Conference on Computer Vision and Pattern Recognition Workshops.

[47] Skibbe H., Reisert M. (2017). Spherical Tensor Algebra: A Toolkit for 3D Image Processing. J. Math. Imaging Vis..

[48] Meesters S., Ossenblok P., Wagner L., Schijns O., Boon P., Florack L., Vilanova A., Duits R. (2017). Stability metrics for optic radiation tractography: Towards damage prediction after resective surgery. J. Neurosci. Methods.

[49] Reisert M., Kiselev V.G. (2011). Fiber Continuity: An Anisotropic Prior for ODF Estimation. IEEE Trans. Med. Imaging.

[50] Prčkovska V., Rodrigues P., Duits R., Haar Romenij B.T., Vilanova A. Extrapolating fiber crossings from DTI data: Can we infer similar fiber crossings as in HARDI?. Proceedings of the Workshop on Computational Diffusion MRI, MICCA.

[51] Iijima T. (1959). Basic Theory of Pattern Observation.

[52] Koenderink J.J. (1984). The structure of images. Biol. Cybern..

[53] ter Haar Romeny B.M. (2003). Front-End Vision and Multi-Scale Image Analysis: Multi-Scale Computer Vision Theory and Applications, Written in Mathematica.

[54] Weickert J. (1998). Anisotropic Diffusion in Image Processing.

[55] Duits R., Burgeth B. (2007). Scale Spaces on Lie Groups. SSVM.

[56] Benoist Y., Quint J.F. (2016). Central limit theorem for linear groups. Ann. Probab..

[57] Pilte M., Bonnabel S., Barbaresco F. Maneuver Detector for Active Tracking Update Rate Adaptation. Proceedings of the 2018 19th International Radar Symposium (IRS).

[58] Berger J., Neufeld A., Becker F., Lenzen F., Schnörr C., Aujol J.F., Nikolova M., Papadakis N. (2015). Second Order Minimum Energy Filtering on SE(3) with Nonlinear Measurement Equations. Scale Space and Variational Methods in Computer Vision.

[59] Oksendal B. (1998). Stochastic Differential Equations.

[60] Hsu E. (2002). Stochastic Analysis on Manifolds.

[61] Feller W. (1966). An Introduction to Probability Theory and Its Applications.

[62] Felsberg M., Duits R., Florack L. (2003). The Monogenic Scale Space on a Bounded Domain and its Applications. Scale Space Methods in Computer Vision. Scale-Space 2003.

[63] Duits R., Felsberg M., Florack L.M.J. (2003). *α* Scale Spaces on a Bounded Domain. Scale Space Methods in Computer Vision. Scale-Space 2003.

[64] Duits R., Florack L., Graaf J.D., Romeny B.T.H. (2004). On the Axioms of Scale Space Theory. J. Math. Imaging Vis..

[65] Pedersen K.S., Duits R., Nielsen M., Kimmel R., Sochen N.A., Weickert J. (2005). On *α* Kernels, Lévy Processes, and Natural Image Statistics. Scale Space and PDE Methods in Computer Vision.

[66] Yosida K. (1980). Functional Analysis.

[67] Winkels M., Cohen T.S. (2018). 3D G-CNNs for Pulmonary Nodule Detection. arXiv.

[68] Worrall D., Brostow G. (2018). CubeNet: Equivariance to 3D Rotation and Translation. arXiv.

[69] Weiler M., Geiger M., Welling M., Boomsma W., Cohen T. (2018). 3D Steerable CNNs: Learning Rotationally Equivariant Features in Volumetric Data. arXiv.

[70] Montobbio N., Sarti A., Citti G. (2018). A metric model for the functional architecture of the visual cortex. arXiv.

[71] Oyallon E., Mallat S., Sifre L. (2013). Generic deep networks with wavelet scattering. arXiv.

[72] Kanti V., Mardia P.E.J. (1999). Directional Statistics.

[73] Wu L., Birge J.R., Linetsky V. (2007). Chapter 3 Modeling Financial Security Returns Using Lévy Processes. Handbooks in Operations Research and Management Science.

[74] Belkic D.D., Belkic K. (2010). Signal Processing in Magnetic Resonance Spectroscopy with Biomedical Applications.

[75] Chirikjian G. (2010). Information-theoretic inequalities on unimodular Lie groups. J. Geom. Mech..

[76] Barbaresco F. (2018). Higher Order Geometric Theory of Information and Heat Based on Poly-Symplectic Geometry of Souriau Lie Groups Thermodynamics and Their Contextures: The Bedrock for Lie Group Machine Learning. Entropy.

[77] Akian M., Quadrat J., Viot M. (1994). Bellman processes. Lect. Notes Control Inf. Sci..

[78] Schmidt M., Weickert J. (2016). Morphological Counterparts of Linear Shift-Invariant Scale-Spaces. J. Math. Imaging Vis..

[79] Hörmander L. (1967). Hypoelliptic second order differential equations. Acta Math..

[80] Misiorek A., Weron R., Gentle J.E., Härdle W.K., Mori Y. (2012). Heavy-Tailed Distributions in VaR Calculations. Handbook of Computational Statistics: Concepts and Methods.

[81] Felsberg M., Sommer G. (2004). The Monogenic Scale-Space: A Unifying Approach to Phase-Based Image Processing in Scale-Space. J. Math. Imaging Vis..

[82] Kanters F., Florack L., Duits R., Platel B., ter Haar Romeny B. (2007). ScaleSpaceViz: *α*-Scale spaces in practice. Pattern Recognit. Image Anal..

[83] Duits R., Franken E. (2010). Left-invariant parabolic evolutions on SE(2) and contour enhancement via invertible orientation scores Part I: Linear left-invariant diffusion equations on SE(2). Q. Appl. Math..

[84] Duits R., Franken E. (2011). Left-Invariant Diffusions on the Space of Positions and Orientations and their Application to Crossing-Preserving Smoothing of HARDI images. Int. J. Comput. Vis..

[85] Duits R., Dela Haije T., Creusen E., Ghosh A. (2012). Morphological and Linear Scale Spaces for Fiber Enhancement in DW-MRI. J. Math. Imaging Vis..

[86] Duits R., Bekkers E., Mashtakov A. (2018). Fourier Transform on the Homogeneous Space of 3D Positions and Orientations for Exact Solutions to PDEs. arXiv.

[87] Portegies J., Sanguinetti G., Meesters S., Duits R., Aujol J.F., Nikolova M., Papadakis N. (2015). New Approximation of a Scale Space Kernel on SE(3) and Applications in Neuroimaging. SSVM.

[88] Arendt W., Bukhvalov A.V. (1994). Integral representation of resolvent and semigroups. Forum Math..

[89] Griffiths D. (1994). Introduction to Quantum Mechanics.

[90] Wigner E. (1959). Gruppentheorie und ihre Anwendungen auf die Quantenmechanik der Atomspektren. Braunschweig: Vieweg Verlag.

[91] Margenau H., Murphy G.M. (1956). The Mathematics of Physics and Chemistry.

[92] ter Elst A.F.M., Robinson D.W. (1998). Weighted Subcoercive Operators on Lie Groups. J. Funct. Anal..

[93] Dong H., Chirikjian G. A Comparison of Gaussian and Fourier Methods for Degenerate Diffusions on *SE*(2). Proceedings of the 2015 IEEE Conference on Decision and Control.

[94] Meesters S.P.L., Sanguinetti G.R., Garyfallidis E., Portegies J.M., Duits R. Fast Implementations of Contextual PDE’S for HARDI Data Processing in DIPY; Abstract. Presented at 24th ISMRM Annual Meeting and Exhibition.

[95] Chirikjian G. (2010). Group Theory and Biomolecular Conformation, I.: Mathematical and computational models. J. Phys. Condens. Matter.

[96] Pinsky M.A. (1976). Isotropic transport process on a Riemannian manifold. Trans. Am. Math. Soc..

[97] Prčkovska V., Andorra M., Villoslada P., Martinez-Heras E., Duits R., Fortin D., Rodrigues P., Descoteaux M., Hotz I., Schultz T. (2015). Contextual Diffusion Image Post-processing Aids Clinical Applications. Visualization and Processing of Higher Order Descriptors for Multi-Valued Data.

[98] Meesters S.P.L., Sanguinetti G.R., Garyfallidis E., Portegies J.M., Ossenblok P., Duits R. Cleaning Output of Tractography via Fiber to Bundle Coherence, a New Open Source Implementation; Abstract. Presented at Organization for Human Brain Mapping Annual Meeting.

[99] Liao M. (2004). Lévy Processes in Lie Groups.

[100] Duits R., Ghosh A., Dela Haije T.C.J., Mashtakov A. (2016). On Sub-Riemannian Geodesics in SE(3) Whose Spatial Projections do not Have Cusps. J. Dyn. Control Syst..

